# Transcriptomic decoding of brain function and cerebral blood flow impairments in first-episode drug-naive patients with major depressive disorder

**DOI:** 10.3389/fpsyt.2025.1692921

**Published:** 2025-10-27

**Authors:** Miaoqian Tu, Zelin Yu, Wenwen Song, Xiaomei Shao, Jingjing Xu, Jiangnan Lin, Maosheng Xu, Zhijian Cao

**Affiliations:** ^1^ Department of Radiology, The First Affiliated Hospital of Zhejiang Chinese Medical University, Zhejiang Provincial Hospital of Chinese Medicine, Hangzhou, China; ^2^ The First School of Clinical Medicine, Zhejiang Chinese Medical University, Hangzhou, China; ^3^ The Third School of Clinical Medicine, Zhejiang Chinese Medical University, Hangzhou, China; ^4^ Department of Neurobiology and Acupuncture Research, The Third School of Clinical Medicine (School of Rehabilitation Medicine), Zhejiang Chinese Medical University, Key Laboratory of Acupuncture and Neurology of Zhejiang Province, Hangzhou, China; ^5^ Department of Radiology, The Second Affiliated Hospital of Zhejiang University School of Medicine, Hangzhou, China

**Keywords:** major depressive disorder, amplitude of low-frequency fluctuations, cerebral blood flow, neurovascular coupling, functional enrichment analysis

## Abstract

**Background:**

Major depressive disorder (MDD) is highly prevalent and severely impacts daily life. The objective of this study is to investigate the genetic mechanisms behind brain function and perfusion abnormalities, measured by the amplitude of low-frequency fluctuations (ALFF) and cerebral blood flow (CBF) in first-episode drug-naive MDD (FEDN-MDD).

**Methods:**

In this study, we analyzed ALFF and CBF alterations in 34 FEDN-MDD patients and 32 matched healthy controls (HCs) using multimodal magnetic resonance imaging (MRI). Combined with the Allen Human Brain Atlas, we performed spatial correlation analysis between neuroimaging and transcriptomic data, followed by gene enrichment analysis to identify gene expression patterns associated with ALFF and CBF alterations in FEDN-MDD.

**Results:**

Compared to HCs, FEDN-MDD patients exhibited decreased ALFF in the right parahippocampal gyrus, elevated CBF in the right middle frontal gyrus, and reduced CBF in the right superior temporal gyrus. Furthermore, these brain function and perfusion changes were spatially associated with the expression of 1,128 and 1,147 genes, respectively. Importantly, the two gene sets demonstrated both shared and distinct features, primarily related to synaptic plasticity, angiogenesis, and neurovascular coupling (NVC).

**Conclusions:**

These findings highlight the correlation between genetic factors and FEDN-MDD, revealing both shared and distinct molecular associations with brain function and perfusion.

## 1 Introduction

Major Depressive Disorder (MDD) is a severe psychiatric disorder associated with significant emotional and cognitive disturbances (1). It affects around 6% of the global population annually (2), leading to significant economic costs and increased demand on healthcare services. Therefore, understanding the underlying biological mechanisms of MDD is crucial for the identification of potential targets and the development of novel therapeutic approaches.

In recent years, researchers have made significant progress in understanding the neural mechanisms of MDD. Neuroimaging evidence reveals that MDD patients exhibit specific brain function changes, mainly involving the default mode network (DMN) and the prefrontal control network (FPN) (3, 4). Resting-state functional magnetic resonance imaging (fMRI) using blood-oxygenation-level-dependent (BOLD) signal changes has shown that MDD patients exhibit altered amplitude of low-frequency fluctuations (ALFF) in several brain regions, such as the caudate nucleus, corpus callosum (5), and precuneus (6). Moreover, arterial spin labeling (ASL) technique has detected distinct patterns of altered relative cerebral blood flow (rCBF) in individuals with MDD (7). Cooper et al. (8) found abnormal rCBF in the right parahippocampal gyrus, thalamus, and insula in MDD. Another study on the relationship between MDD and CBF also reported notable rCBF changes in the frontal cortex, cingulate cortex, and temporal lobe (9). These studies suggest that MDD patients may simultaneously have abnormal neuronal activity and brain perfusion. However, existing studies are mostly limited to single-modal imaging analysis, making it difficult to integrate multidimensional information regarding neuronal activity and brain perfusion. To better understand the pathophysiological mechanisms underlying brain function and CBF abnormalities in MDD, it is essential to explore their potential molecular and genetic basis.

In recent decades, researchers have invested substantial time and resources into studying MDD, widely believing its onset is closely linked to genetic factors (10, 11). Genome-wide association studies (GWAS) have found that MDD is affected by a range of genes and gene sets (12). The advent of whole-brain gene expression atlases has given rise to a new field of neuroimaging-transcriptomics, enabling the linkage of molecular pathways to macroscopic brain phenotypes. For example, the Allen Human Brain Atlas (AHBA) (13) allows researchers to map gene expression patterns to neuroimaging biomarkers. By means of this powerful approach, researchers have discovered the correlation between neuroimaging results and transcriptomics in MDD (7), suggesting that altered CBF in MDD may be associated with gene expression. However, most studies have focused on patients after drug treatment, ignoring the potential impact of medication on the results. It remains unclear whether these gene-level evidences are related to specific brain functions and perfusion abnormalities in early-stage MDD. Therefore, investigating the molecular basis of brain function and perfusion abnormalities in first-episode drug-naive MDD (FEDN-MDD) patients is crucial for understanding the early stages of MDD.

**Figure 1:**
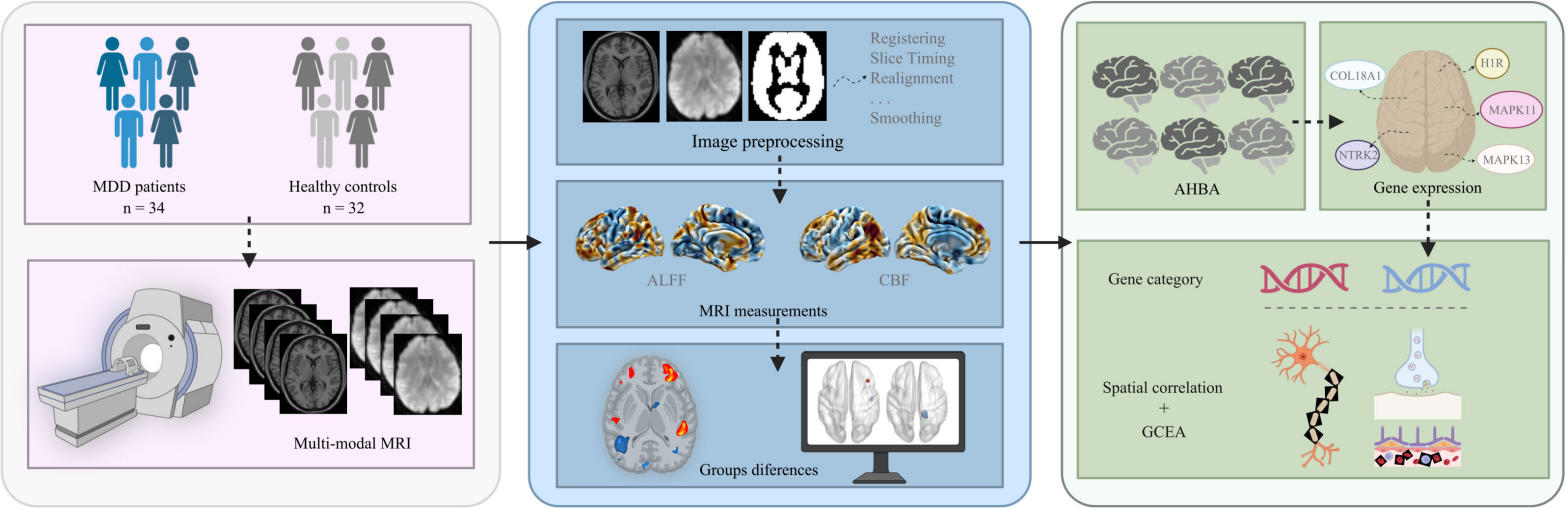
The study design and analysis pipeline. MDD, major depressive disorder; MRI, magnetic resonance imaging; ALFF, amplitude of low-frequency fluctuations; CBF, cerebral blood flow, AHBA; Allen Human Brain Atlas; GCEA, gene category enrichment analysis. Figure created with BioRender.com (URL).

In this study, we selected FEDN-MDD patients and applied multi-modal MRI. In combination with the AHBA, a neuroimaging-transcriptomics association analysis was performed to explore the molecular mechanisms underlying brain function and perfusion changes in FEDN-MDD from multiple dimensions, aiming to identify potential biomarkers and therapeutic targets for early intervention. The study design and analysis pipeline is outlined in a schematic form in **Figure 1**.

## 2 Methods

### 2.1 Participants

Initially, 82 right-handed individuals participated in this study. 16 participants were excluded due to poor image qualities. Finally, 66 participants were included (mean age 23.0 ± 6.5 years), comprising 34 FEDN-MDD patients and 32 HCs. Neuropsychological tests were evaluated, and all participants completed the resting-state fMRI and ASL scans. FEDN-MDD patients were recruited from the outpatient Mental Health Departments of the First Affiliated Hospital, Xinhua Hospital, and Zhongshan Hospital, affiliated to Zhejiang Provincial Hospital of Traditional Chinese Medicine, between July 2022 and December 2024. The diagnosis of each FEDN-MDD patient was confirmed by two experienced mental health physicians according to the International Classification of Diseases, 10th Revision (ICD-10) diagnostic criteria for MDD. Matched for handedness, age, and sex, the HCs were recruited through community and media advertisements. It is critical to note that all MRI scans for both FEDN-MDD patients and HCs were conducted exclusively at the Hubin Campus of Zhejiang Provincial Hospital of Traditional Chinese Medicine, using the same 3.0T GE Discovery MR750 scanner and a standardized imaging protocol, with all scans performed by the same radiologist. This consistent data acquisition procedure effectively eliminated any potential inter-scanner variability, ensuring uniformity across all scans. The Zhejiang Provincial Hospital of Traditional Chinese Medicine’s ethics committee approved this study (Ethics No. 2022-KL-177-01). All participants voluntarily signed a written informed consent form before the study began.

### 2.2 Inclusion and exclusion criteria

Inclusion criteria for FEDN-MDD patients: 1) MDD diagnosed by two experienced psychiatrists according to the ICD-10. 2) Hamilton Depression Scale (HAMD) -17 score > 7; 3) no history of any form of medical treatment; 4) between 14–59 years; 5) right-handed; 6) with no conflicting MRI findings. Exclusion criteria: 1) other major mental illnesses or neurodegenerative diseases; 2) history of traumatic brain injury; 3) alcohol or drug abuse history; 4) uncontrolled primary diseases (cardiovascular, respiratory, hematological, endocrine, etc.); 5) participation in other clinical trials within the past month; 6) MRI contraindications.

Inclusion criteria for HCs: Mini-Mental State Examination (MMSE) score ≥ 26 and HAMD-17 score ≤ 7. Exclusion criteria for the HC group were similar to those of the FEDN-MDD group.

### 2.3 Imaging acquisition

All participants underwent brain MRI, which was performed with 3.0-Tesla General Electric scanners (Discovery MR750, GE Healthcare) equipped with an 8-channel head coil at Zhejiang Provincial Hospital of Traditional Chinese Medicine. Before the scan, instruct the patient to lie on their back, relax, remain still, close their eyes, and avoid thinking or falling asleep. The operator used tight sponge pads to reduce head movement of the subjects, and used earplugs to minimize the impact of MRI scanner noise on them. In this study, each participant underwent the acquisition of the following multi-modal MRI data: T1-weighted sagittal structural images, BOLD-fMRI images, cross-sectional T2-weighted images, ASL images, and CBF images automatically generated from the ASL images. Detailed scanning parameters can be found in the **Supplementary Data 1**.

### 2.4 Image preprocessing

Two experienced radiologists assessed image quality to ensure that there were no obvious head movement artifacts or abnormal signals, and then classified and processed the scanned data. Magnetic resonance data were preprocessed using the DPABI toolbox (http://rfmri.org/dpabi) (14) and SPM8.0 (http://www.fil.ion.ucl.ac.uk/spm) in MATLAB 2020a, mainly includes the following steps: 1) convert DICOM format data to NII or NIFTI formats; 2) remove the first 10 time points to reduce the initial instability of the magnetic field and the impact of the subjects’ discomfort with image acquisition; 3) slice timing, which corrects for data bias introduced by MR scans; 4) realign, head movement files were checked, and subjects with head movement displacement ≤ 3 mm or head movement Angle ≤ 3° were retained; 5) Spatial normalization was performed by segmenting the images and normalizing them to the standard MNI space; 6) regression covariates to eliminate the influence of head movement, cerebrospinal fluid, and white matter signals; 7) a temporal band-pass filter between 0.01-0.10 Hz after regression was used to minimize the influence of heartbeat, breathing signals and the noise of the scanner itself; 8) smooth. After the above steps, the data of 66 subjects met the requirements and were included in this study.

### 2.5 ALFF analysis

The previously preprocessed results were used for ALFF analysis, based on the ALFF module in the DPABI toolbox. ALFF is a common fMRI metric. It is based on the fast Fourier transform of each voxel signal of the time series in the whole brain image and converted to the square root of the power spectrum in the frequency domain. In this study, ALFF represents the mean value of each voxel in the frequency range of 0.01 - 0.10 Hz.

### 2.6 CBF analysis

Obtain the CBF images automatically generated based on ASL sequences for all subjects, convert them to NII format using the dcm2nii toolbox in the Mricron, and check the image quality. Subsequently, CBF images were registered to PET templates, segmented, and spatially normalized to Montreal Neurological Institute (MNI) space using SPM8.0. After checking the image quality again, the voxel size was resampled to 2 mm × 2 mm × 2 mm. Data normalization of CBF images for each subject was performed using a z-score, the CBF value for each voxel was subtracted from the whole-brain mean and divided by the standard deviation within a whole-brain mask, and finally divided into zCBF maps for each subject. An 8 mm × 8 mm × 8 mm Full Width at Half Maximum (FWHM) Gaussian kernel was applied to spatially smooth the zCBF maps to optimize the whole-brain statistical analysis.

### 2.7 Transcriptomic analysis

The gene expression data were taken from the AHBA (http://www.brain-map.org) (15–17). This atlas contains data from six postmortem human brains, covering more than 20,000 genes measured in 3,702 different brain areas (**Supplementary Table 1**). And it enables the analysis of spatial associations between brain function and perfusion changes (e.g., ALFF and CBF in FEDN-MDD) and regional gene expression patterns. We preprocessed the raw expression data of over 20,000 genes using the abagen toolbox (18), which provides a standardized workflow to ensure the reproducible processing of gene expression data in accordance with established guidelines (16).

After preprocessing gene expression data using the abagen toolbox in combination with the 636-region integrated human brain network atlas. This atlas includes the first 600 regions from Schaefer et al.’s 17-network multiscale parcellation system based on resting-state functional connectivity (**Supplementary Table 2**), and the remaining 36 regions [211-246] from the Brainnetome Atlas (https://atlas.brainnetome.org/). Subsequently, we constructed a regional expression matrix for each donor (636 × 15,633), where the rows corresponding to brain regions and the columns correspond to retained genes. To select stably expressed genes, we calculated the average similarity across brain regions between donors (threshold > 0.1), ultimately retaining 11,803 genes. Subsequently, we averaged the expression levels of the retained genes across all donors, ultimately generating a 636 × 11,803 matrix that describes the transcriptomic levels of the entire cortical brain.

Partial least squares (PLS) correlation analysis (19) was used to validate the neuroimaging-transcriptomic correlations of ALFF and CBF in FEDN-MDD patients. The specific steps are detailed in **Supplementary Data 2**.

### 2.8 Gene enrichment analysis

A set of enrichment analyses was for the identified genes associated with brain ALFF and CBF changes in FEDN-MDD patients. First, systematic functional annotation was carried out using the DAVID bioinformatics analysis platform (v2023q3) (20). Gene Ontology (GO) was used to analyze biological processes (BPs), molecular functions (MFs), and cellular components (CCs), while the Kyoto encyclopedia of genes and genome (KEGG) pathway database was used to identify significantly enriched biological pathways.

### 2.9 Statistical analysis

#### 2.9.1 Demographic and clinical data

The differences in age, clinical scale scores and mean FD (framewise displacement). between the two groups were analyzed using a two-sample t-test. A *χ* ^2^-test was used to compare intergroup sex differences. *P*-value < 0.05 was considered statistically significant, and statistical analyses were performed using the SPSS software package (version 22.0).

#### 2.9.2 ALFF and CBF analysis

Two-sample t-tests of ALFF and CBF values were performed using DPABI, with sex, age, and mean FD included as covariates. Multiple comparisons were corrected using Gaussian Random Field (GRF) theory with a voxel-level threshold of *P* < 0.001 and a cluster-level threshold of *P* < 0.05. Significant results and corresponding brain regions were visualized with BrainNet Viewer (Version 1.7) (21).

#### 2.9.3 Transcriptomics

The statistical tests, sample size, number of repetitions, and the definition of repetitions used in the transcriptomics-neuroimaging spatial correlation analysis and gene enrichment analysis are described in the corresponding methods. Statistical significance was defined as two-sided *P* < 0.05, and multiple comparisons were controlled for using the Benjamini–Hochberg procedure for false discovery rate (FDR) correction.

## 3 Results

### 3.1 Demographic and clinical characteristics

A total of 66 participants were included in this study. The FEDN-MDD group consisted of 34 participants, including 8 males and 26 females, with ages ranging from 17 to 38 years (24.59 ± 5.14). The HC group included 32 participants, with 8 males and 24 females, aged 14 to 38 years (21.59 ± 7.27). There were no significant group differences in gender (*P* = 0.635) and age (*P* = 0.112). However, significant differences were observed in clinical scores (*P* < 0.001). Compared to the HC group, the FEDN-MDD group had significantly higher depression and anxiety scores (*P* < 0.001) (**Table 1**).

**Table 1:** Demographics and clinical characteristics of the participants.

Sample size	FEDN-MDD (n = 34)	HCs (n = 32)	t value or x^2^ value	*P* value
Age (years)	24.59 ± 5.14	21.59 ± 7.27	1.899	0.112
Sex (male/female)	8/28	6/26	0.225	0.635
HAMD (scores)	23.44 ± 7.60	2.00 ± 2.10	-15.831	<0.001
HDMD (scores)	18.29 ± 7.75	2.28 ± 2.82	-11.278	<0.001
Mean FD (mm)	0.62 ± 0.31	0.51 ± 0.16	-1.794	0.078

*P < 0.05 is considered statistically significant.

FEDN-MDD, first-episode drug-naive major depressive disorder; HCs, healthy controls; SD, standard deviation; HAMD, Hamilton Depression Rating Scale; HDMD, High-Density Magnetic Data; FD, framewise displacement.

### 3.2 ALFF results

**Figure 2:**
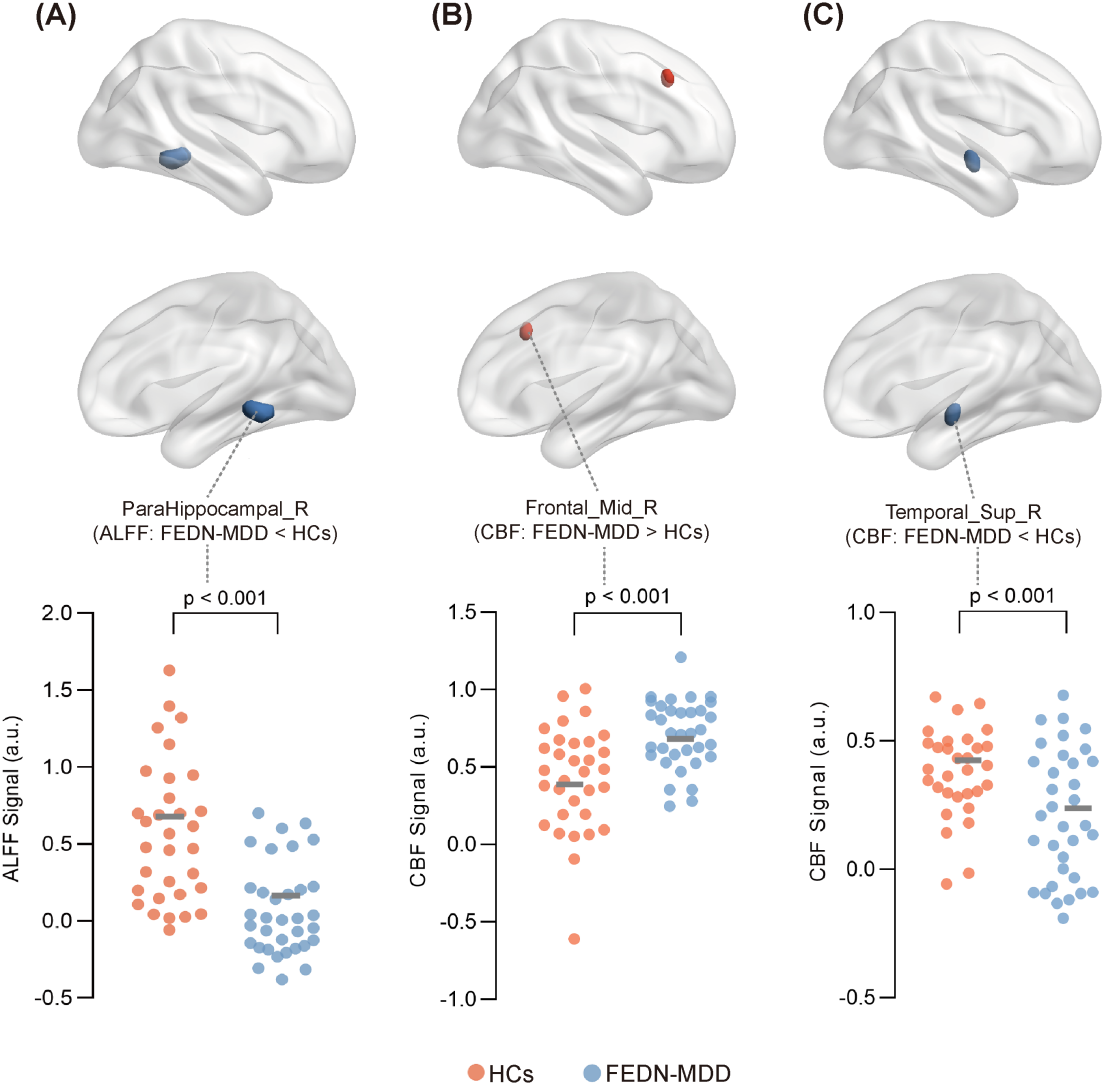
Significant differences in ALFF or CBF between FEDN-MDD group and HCs.Surface plot showing the spatial locations of brain regions with significant differences in ALFF or CBF between the two groups, along with the ALFF or CBF values extracted from these regions for all participants. In the brain region spatial maps, red and blue represent regions with elevated and reduced ALFF/CBF, respectively. In the scatter plots, orange circles represent the HCs, and blue circles denote the FEDN-MDD patients. Compared to the HCs, FEDN-MDD group showed decreased ALFF in the right parahippocampal gyrus **(A)**, increased CBF in the right middle frontal gyrus **(B)**, and decreased CBF in the right superior temporal gyrus **(C)**. ALFF, amplitude of low-frequency fluctuation; CBF, cerebral blood flow; FEDN-MDD, first-episode drug-naive patients with major depressive disorder; HCs, healthy controls; R, right.

In the FEDN-MDD group, only the ALFF value in the right parahippocampal gyrus was decreased, compared to that in the HC group (voxel-level *P* < 0.001, cluster-level *P* < 0.05, GRF corrected) (line 1 of **Table 2**, **Figure 2**).

**Table 2:** Brain regions with ALFF and CBF changes in FEDN-MDD patients compared to HCs.

Brain region	Cluster voxels	Peak intensity	Peak MNI (mm)		
			X	Y	Z
Parahippocampal gyrus_R	28	-5.0123	15	-39	-9
Middle frontal gyrus_R	28	4.1267	38	24	40
Superior temporal gyrus_R	36	-4.24266	46	-12	-10

MNI, Montreal Neurological Institute; R, right.

### 3.3 CBF results

Compared to the HCs, the FEDN-MDD patients showed increased CBF in the right middle frontal gyrus and decreased CBF in the right superior temporal gyrus (voxel-level *P* < 0.001, cluster-level *P* < 0.05, GRF corrected) (line3–4 of **Table 2**, **Figure 2**).

### 3.4 Genes associated with brain ALFF and CBF changes in FEDN-MDD

**Figure 3:**
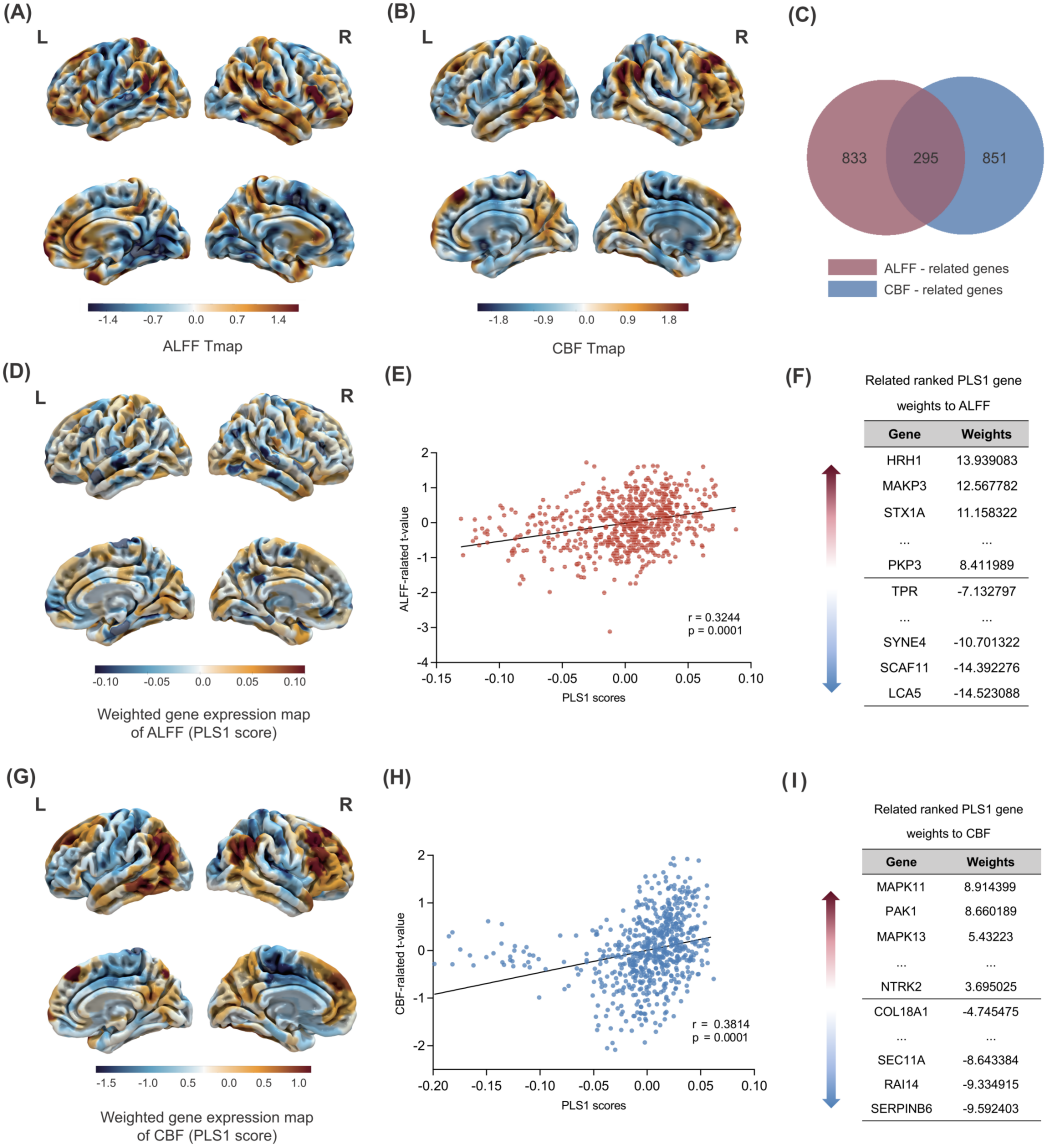
Gene PLS analysis and enrichment analyses. Changes in ALFF across the whole brain in FEDN-MDD. **(A)** Changes in ALFF across the whole brain. **(B)** Changes in CBF across the whole brain. **(C)** Intersection analysis of the ALFF-related gene set and the CBF-related gene set. **(D)** Spatial map of the whole-brain PLS1 scores related to ALFF.**(E)** The spatial correlation analysis between PLS1 scores and brain ALFF changes in FEDN-MDD. **(F)** The ranked gene weights for PLS1 associated with ALFF changes in FEDN-MDD (*P* < 0.05). **(G)** Spatial map of the whole-brain PLS1 scores related to CBF. **(H)** The spatial correlation analysis between PLS1 scores and brain CBF changes in FEDN-MDD. **(I)** The ranked gene weights for PLS1 associated with CBF changes in FEDN-MDD (*P* < 0.05). The statistical maps (A, B, D, and G) reflect the contrast of FEDN-MDD > HC. In these maps, warm colors (orange/red) represent regions with significant increases in the FEDN-MDD group compared to the healthy control group (i.e., FEDN-MDD > HC), while cool colors (blue) represent regions with significant decreases in the FEDN-MDD group compared to the healthy control group (i.e., FEDN-MDD < HC).

By leveraging transcriptomics-neuroimaging spatial correlation analysis, we found that brain ALFF and CBF changes in FEDN-MDD patients were associated with expression levels of 1128 (**Figure 3F**) and 1147 genes (**Figure 3I**), respectively (*P* < 0.05), with 294 overlapping genes (**Figure 3C**). The spatial correlation tests demonstrated that the results of our brain ALFF (r = 0.3244, *P* = 0.0001) and CBF (r = 0.3814, *P* = 0.0001) analyses were significantly different from random (**Figure 3D, E, G, H**).

### 3.5 Gene functional enrichment

**Figure 4:**
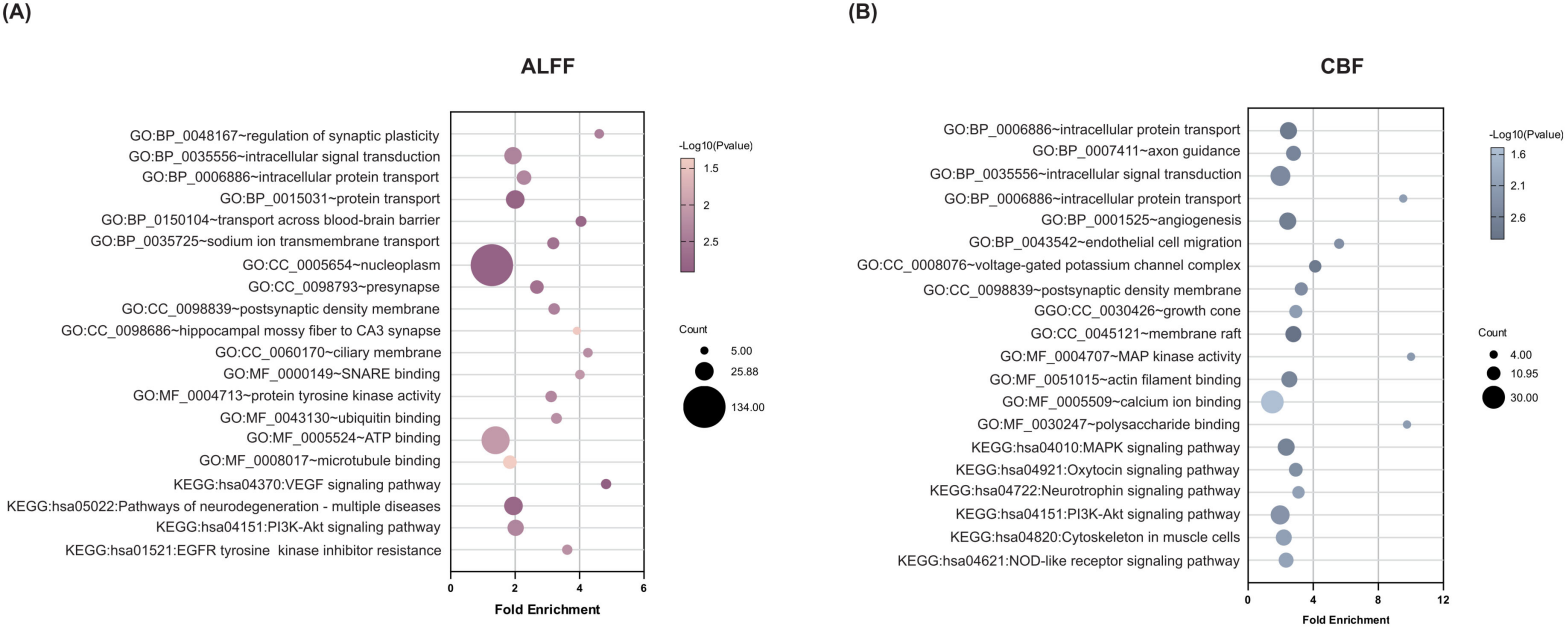
Functional enrichment of genes associated with brain ALFF and CBF changes in FEDN-MDD. A combination of transcription-neuroimaging spatial correlation and the ensemble based GCEA revealed that ALFF **(A)** and CBF **(B)** changes were correlated with MDD-general GO categories. The x-axis represents the fold enrichment and the y-axis shows the iterms from the GO and pathway databases. The bubble size indicates the number of genes overlapping with each iterm, and the bubble color represents -log10(p-value). ALFF, amplitude of low-frequency fluctuations; CBF, cerebral blood flow; GO, gene ontology; BP, biological process; CC, cellular component; MF, molecular function; KEGG, Kyoto encyclopedia of genes and genome; MAPK, mitogen-activated protein kinase; VEGF, vascular endothelial growth factor; GCEA, gene category enrichment analysis.

To clarify the biological functions and pathway characteristics of gene sets associated with ALFF and CBF alterations in FEDN-MDD, we performed functional enrichment analysis using the DAVID database (**Figure 4**). Overall, the two gene sets exhibited both shared and unique functional enrichment results. First, both gene sets were enriched for shared BPs, including intracellular signal transduction and intracellular protein transport. The ALFF-related gene set was enriched in unique BPs, including regulation of synaptic plasticity, protein transport, blood-brain barrier (BBB) substance transport, among others; the CBF-related gene set was specifically enriched in unique BPs, including axon guidance, angiogenesis, among others. Second, the CCs analysis further revealed that both gene sets were enriched in shared CCs, particularly in the postsynaptic density. The ALFF-related gene set was specifically enriched in structures such as the presynapse, ciliary membrane, among others; the CBF-related gene set was specifically enriched in structures such as the voltage-gated potassium channel complex, growth cone, among others; Third, the MFs analysis revealed no significant common abnormalities between the two gene sets. Notably, the ALFF-related gene set was enriched in unique MFs, SNARE binding, protein tyrosine kinase activity, and ATP binding, among others; the CBF-related gene set was enriched in distinct MFs, including MAP kinase activity, actin filament binding, calcium ion binding, among others. Finally, at the KEGG pathway level, both gene sets were co-enriched in the PI3K-Akt signaling pathway. The ALFF-related gene set was enriched in unique pathways, including VEGF signaling pathways, neurodegenerative disease, and others; the CBF-related gene set was enriched for unique pathways such as MAPK signaling pathway, neurotrophin signaling pathway, oxytocin signaling pathway, etc.

## 4 Discussion

This study is the first to investigate the genetic mechanisms underlying brain functional changes and blood flow perfusion impairment in FEDN-MDD by combining multi-modal MRI analysis with gene expression data. The multi-modal MRI analysis revealed that the differences in ALFF and CBF between FEDN-MDD and HCs were primarily concentrated in the DMN, particularly in the parahippocampal gyrus and superior temporal gyrus, as well as in the FPN, specifically the middle frontal gyrus. Furthermore, these brain function and perfusion changes were spatially associated with expression levels of 1128 and 1147 genes, respectively. Importantly, both commonalities and differences in the two gene sets and their functional characteristics. Overall, the present study enhances the understanding of abnormal brain activity patterns and their molecular mechanisms in FEDN-MDD patients.

FEDN-MDD patients showed significantly reduced ALFF in the right parahippocampal gyrus, suggesting reduced neuronal activity and functional impairment in this brain region during resting state. The parahippocampal gyrus is a key brain region in the DMN, primarily involved in the memory and emotion integration circuitry (22). Disruption of the default mode network is closely linked to core clinical symptoms of MDD, such as rumination and memory integration deficits (3, 23). Chronic stress has been shown to impair synaptic plasticity in the parahippocampal gyrus (24), consistent with our finding that this functional alteration was spatially coupled with gene expression profiles enriched for synaptic plasticity pathways (e.g., H1R), thereby offering a molecular framework for the observed neuronal dysfunction.

Our CBF findings in FEDN-MDD revealed a dissociative pattern: hyperperfusion in the right middle frontal gyrus (a FPN node) and hypoperfusion in the right superior temporal gyrus (a DMN node). This pattern aligns with the “dual-network imbalance” model of MDD (25), characterized by excessive FPN engagement and insufficient DMN suppression, and provides a novel hemodynamic perspective on early-stage MDD. Unlike the prefrontal hypometabolism observed in chronic MDD (26), the frontal hyperperfusion in our drug-naïve patients may reflect early compensatory recruitment or glutamatergic overactivity (27), whereas the temporal hypoperfusion may indicate impaired DMN regulation.

Based on the emerging “transcriptional vulnerability” hypothesis (28), we conducted a spatial correlation analysis between transcriptomic data and neuroimaging features in FEDN-MDD patients. A total of 1,128 genes were linked to ALFF alterations and 1,147 to CBF changes, with 294 genes common to both. This finding is consistent with the view that the complex neurophenotypes of FEDN-MDD arise from the combined effects of multiple genes, consistent with previous GWAS evidence (29).

Functional enrichment analysis revealed the molecular mechanisms underlying the neuroimaging phenotypes in FEDN-MDD patients. Gene sets associated with ALFF and CBF changes exhibited both shared and unique biological functions and pathways, reflecting functional coordination and imbalance at the neurovascular unit (NVU) level. In terms of synaptic signaling and plasticity, both gene sets were jointly enriched in the postsynaptic density (*P* = 0.004 for both), a key structure involved in neural signal transmission. The ALFF-related gene set was specifically enriched in the regulation of synaptic plasticity(*P* = 0.004), with H1R (z-score = 13.939) identified as the key regulator of this process. Previous studies have shown that abnormal expression of H1R receptors in the central nervous system of MDD patients is associated with symptoms such as low mood and sleep disturbances (30). Additionally, neuroimaging evidence shows reduced H1R binding in the prefrontal, frontal, and cingulate cortices in MDD, negatively correlating with depressive symptom severity (31). In parallel, the CBF-related gene set was specifically enriched in the neurotrophin signaling pathway (*P* = 0.004), driven by key genes such as NTRK2 (z-score = 3.695), MAPK11 (z-score = 8.914), and MAPK13 (z-score = 5.432). NTRK2 (TrkB receptor) serves as a major signaling molecule for synaptic plasticity (32, 33). Earlier research reported reduced TrkB concentrations in the brains of MDD patients (34). MAPK11/13 (p38 MAPK isoforms) also play a critical role in synaptic plasticity, and extensive research have demonstrated that their altered activity is closely linked to the pathological processes of MDD (35, 36). In terms of angiogenesis, vascular remodeling, and the BBB, the ALFF-related gene set was significantly enriched in the VEGF signaling pathway (*P* = 0.001), with the key regulatory gene MAPK3 (ERK1) (z-score = 12.568) closely linked to the pathogenesis, pathological features, and treatment response of depression through its signaling cascade (37, 38). In addition, the CBF-related gene set was significantly enriched in angiogenesis (*P* = 0.002), with COL18A1 (z-score = - 4.475) identified as a key regulator. COL18A1 is crucial for maintaining basement membrane integrity, and its disruption may impair the BBB function. In patients with cerebral small vessel disease (CSVD), COL18A1 is markedly upregulated in the vasculature (39), pointing to a potential link between vascular remodeling and CBF regulation in MDD. Importantly, both gene sets were significantly enriched in the PI3K-Akt signaling pathway (ALFF gene set *P* = 0.002, CBF gene set *P* = 0.005). As a core component of NVC, the PI3K-Akt pathway simultaneously regulates synaptic plasticity (40) and angiogenesis (41). Dysregulation of this pathway has been extensively linked to the pathophysiology of neuropsychiatric conditions such as MDD and epilepsy (40, 42). Our findings show that CBF-related gene set was significantly enriched in synaptic plasticity pathways, implying that abnormal neuronal activity may influence blood flow. Meanwhile, the ALFF-related gene set was enriched in pathways related to vascular function, indicating that the vascular microenvironment may influence neuronal activity. The shared enrichment of the PI3K-Akt signaling pathway in both gene sets points to a potential molecular association with NVC dysregulation in FEDN-MDD, indicating a disruption in the integration of neuronal activity and blood flow at the NVU level.

Although neuronal activity and perfusion are closely linked through NVC, their relationship is not linear. In this study, we observed reduced ALFF in the right parahippocampal gyrus (DMN node) of FEDN-MDD patients, reflecting suppressed neuronal activity, but no significant CBF change in the same region. Similarly, increased CBF in the right middle frontal gyrus (FPN node) and decreased CBF in the right superior temporal gyrus (DMN node) did not match ALFF changes. The dissociation between ALFF and CBF findings may reflect two factors. The first is their distinct physiological roles: ALFF reflects spontaneous neuronal activity (43), while CBF indicates the vascular support of neuronal energy needs (44). The second is the possibility that, under pathological conditions, the coupling between neural activity and blood flow becomes selectively impaired. Our transcriptomic analysis supports this, showing that while ALFF and CBF are spatially dissociated in neuroimaging, their associated gene sets were enriched in synaptic plasticity and angiogenesis pathways, converging on the PI3K-Akt signaling pathway. This suggests a shared molecular basis underlying neuronal and vascular functions. Notably, this phenomenon of neurovascular uncoupling has also been reported in the early stages of other neurological disorders. For example, in Alzheimer’s disease (AD), a mismatch between neuronal activity and cerebral blood flow occurs in specific brain regions (45). Neuronal activity increases metabolic demand, influencing local blood flow, a process involving multiple cell types and molecular signals, reflecting the complexity of neurovascular regulation (46). Thus, the observed differences are not contradictory but are indicative of the layered complexity of neuronal, vascular, and molecular mechanisms involved in early-stage depression.

Several limitations are worth mentioning in this study. First, the gender imbalance (male:female = 14:52) may limit the generalizability of our findings, given the established sex differences in MDD neurobiology. Although no statistical difference in gender distribution was observed between the groups, the relatively high proportion of female participants suggests that our findings may be more representative of the female FEDN-MDD population. Future studies with larger, gender-balanced samples are essential to validate the robustness of our results and explore potential sex-specific pathophysiological mechanisms. Second, age-based subgroup analysis (e.g., adults vs. minors) was not performed in this study. However, age-related differences in the etiology and neurobiological mechanisms of depression may influence the interpretation of neuroimaging-transcriptomic spatial associations. In future studies, we will increase the sample size and perform more detailed age-stratified analyses to further validate the reliability of these results. Finally, the gene expression data in this study were derived from six postmortem human brains, while the neuroimaging data were collected from the brains of living subjects, introducing a difference in sample sources. Previous studies have shown that human gene expression profiles exhibit high conservatism across individuals (15, 47). To ensure the feasibility of the transcriptome-neuroimaging spatial correlation analysis, we set a differential expression threshold (> 0.1) and selected genes with relatively conserved expression patterns for further analysis. However, this approach may overlook some genes with important biological significance that exhibit greater expression variability across individuals.

## 5 Conclusion

This study proposes a multi-level “molecule-synapse-vascular-network” framework for FEDN-MDD. Our findings provide substantial evidence for the molecular mechanisms of altered brain function and perfusion in FEDN-MDD, offering new insights and identifying valuable targets for future therapeutic development.

## Data Availability

The raw data supporting the conclusions of this article will be made available by the authors, without undue reservation.
