# Supplementary material for: Transcriptomic decoding of brain function and cerebral blood flow impairments in first-episode drug-naive patients with major depressive disorder
Source: Front Psychiatry. 2025 Oct 27;16:1692921. doi: 10.3389/fpsyt.2025.1692921 (PMC12597995; doi:10.3389/fpsyt.2025.1692921)
Supplement: Supplementary file 1 [file DataSheet1.docx]

Supplementary Material

# Supplementary data

# **1.1** Supplementary data 1. **Detailed scanning parameters**

Magnetization-prepared rapid gradient echo sequence was used to acquire high-resolution 3D T1-weighted sagittal images. The parameters were as follows: repeat time (TR) = 8.2 ms; echo time (TE) = 3.2 ms; flip angle (FA) = 12°; matrix size = 256 mm × 256 mm; slice thickness = 1 mm; 152 slices. Resting-state BOLD fMRI data were collected using a gradient-echo single-shot echo planar imaging sequence with the following parameters: TR = 2000 ms; TE = 35 ms; FA = 90°; matrix size = 64 mm × 64 mm; slice thickness = 5 mm, 6960 slices; Cross-sectional T2-weighted images were acquired to exclude organic brain lesions with the following parameters: TR = 5813.5 ms; TE = 84.1 ms; slice thickness = 4 mm, 20 slices; The parameters of ASL were as follows: TR = 5029 ms, FA = 90°, matrix size =128 mm ×128 mm, slice thickness = 4 mm, 72 slices, and cerebral blood flow (CBF) generated automatically from ASL images.

**1.2 Supplementary data 2. Partial least squares (PLS) correlation analysis steps.**

The specific steps are as follows: 1) Spatial resampling was performed on the ALFF Tmap and CBF Tmap of FETN-MDD patients, mapping them to 636 brain regions. The value for each brain region was defined as the mean of all voxel values with in that region. 2) Gene expression data were used as predictor variables, and ALFF Tmap and CBF Tmap were used as the response variables in the PLS regression model. 3) The first component of the PLS model (PLS1) was strongly correlated with ALFF Tmap (r = 0.3244, *P* < 0.001) and CBF Tmap (r = 0.3814, *P* < 0.001) across 636 brain regions. 4) To correct for spatial autocorrelation, we applied the Moran spectral randomization method(1, 2) and performed 5,000 permutation tests on the response variables to assess the statistical significance of the model's explained variance. 5) A bootstrapping method was used to estimate each gene’s weighting coefficient in the PLS analysis. The ratio of the weight of each gene to its bootstrap standard error (5000 times) was utilized to calculate z-scores of each gene weight on PLS1(3). 6) Genes were ranked based on the absolute value of their z-scores to determine their contribution.

**Reference**

1. Wagner HH, Dray S, O'Hara RB. Generating Spatially Constrained Null Models for Irregularly Spaced Data Using Moran Spectral Randomization Methods. Methods in Ecology and Evolution (2015) 6(10):1169-78. doi: 10.1111/2041-210x.12407.

2. Vos de Wael R, Benkarim O, Paquola C, Lariviere S, Royer J, Tavakol S, et al. Brainspace: A Toolbox for the Analysis of Macroscale Gradients in Neuroimaging and Connectomics Datasets. Commun Biol (2020) 3(1):103. Epub 20200305. doi: 10.1038/s42003-020-0794-7.

3. Whitaker KJ, Vértes PE, Romero-Garcia R, Váša F, Moutoussis M, Prabhu G, et al. Adolescence Is Associated with Genomically Patterned Consolidation of the Hubs of the Human Brain Connectome. Proc Natl Acad Sci U S A (2016) 113(32):9105-10. Epub 20160725. doi: 10.1073/pnas.1601745113.

# Supplementary Tables

## Supplementary Table 1. **Demographic information of the six adult donors in the AHBA**

| **Donor** | **Age (years)** | **Sex** | **Ethnicity** | **Hemisphere** | **Post-mortem interval (h)** |
| --- | --- | --- | --- | --- | --- |
| H0315.2001 | 24 | Male | African American | Both | 23 |
| H0315.2002 | 39 | Male | African American | Both | 10 |
| H0315.1009 | 57 | Male | Caucasian | Left | 25.5 |
| H0315.1012 | 31 | Male | Caucasian | Left | 17.5 |
| H0315.1015 | 49 | Female | Hispanic | Left | 30 |
| H0315.1016 | 55 | Male | Caucasian | Left | 18 |

Abbreviations: AHBA, Allen Human Brain Atlas.

## Supplementary Table 2. **Schaefer 2018_600Parcels_17Networks_order**

| **ROI Label** | **ROI Name** | **R** | **A** | **S** |
| --- | --- | --- | --- | --- |
| 1 | 17Networks_LH_VisCent_Striate_1 | -7 | -94 | -14 |
| 2 | 17Networks_LH_VisCent_Striate_2 | -8 | -102 | -2 |
| 3 | 17Networks_LH_VisCent_Striate_3 | -6 | -95 | 1 |
| 4 | 17Networks_LH_VisCent_ExStr_1 | -34 | -59 | -17 |
| 5 | 17Networks_LH_VisCent_ExStr_2 | -35 | -77 | -15 |
| 6 | 17Networks_LH_VisCent_ExStr_3 | -25 | -58 | -9 |
| 7 | 17Networks_LH_VisCent_ExStr_4 | -23 | -73 | -10 |
| 8 | 17Networks_LH_VisCent_ExStr_5 | -41 | -82 | -15 |
| 9 | 17Networks_LH_VisCent_ExStr_6 | -19 | -86 | -15 |
| 10 | 17Networks_LH_VisCent_ExStr_7 | -43 | -86 | -5 |
| 11 | 17Networks_LH_VisCent_ExStr_8 | -25 | -97 | -11 |
| 12 | 17Networks_LH_VisCent_ExStr_9 | -45 | -76 | 4 |
| 13 | 17Networks_LH_VisCent_ExStr_10 | -35 | -90 | 2 |
| 14 | 17Networks_LH_VisCent_ExStr_11 | -20 | -100 | 5 |
| 15 | 17Networks_LH_VisCent_ExStr_12 | -28 | -92 | 10 |
| 16 | 17Networks_LH_VisCent_ExStr_13 | -7 | -97 | 15 |
| 17 | 17Networks_LH_VisCent_ExStr_14 | -37 | -87 | 18 |
| 18 | 17Networks_LH_VisCent_ExStr_15 | -18 | -94 | 20 |
| 19 | 17Networks_LH_VisCent_ExStr_16 | -28 | -84 | 20 |
| 20 | 17Networks_LH_VisCent_ExStr_17 | -23 | -84 | 28 |
| 21 | 17Networks_LH_VisPeri_StriCal_1 | -7 | -62 | 3 |
| 22 | 17Networks_LH_VisPeri_StriCal_2 | -3 | -70 | 5 |
| 23 | 17Networks_LH_VisPeri_StriCal_3 | -6 | -86 | 0 |
| 24 | 17Networks_LH_VisPeri_StriCal_4 | -10 | -73 | 9 |
| 25 | 17Networks_LH_VisPeri_StriCal_5 | -2 | -82 | 11 |
| 26 | 17Networks_LH_VisPeri_ExStrInf_1 | -20 | -49 | -7 |
| 27 | 17Networks_LH_VisPeri_ExStrInf_2 | -12 | -62 | -4 |
| 28 | 17Networks_LH_VisPeri_ExStrInf_3 | -7 | -76 | -6 |
| 29 | 17Networks_LH_VisPeri_ExStrInf_4 | -13 | -42 | -5 |
| 30 | 17Networks_LH_VisPeri_ExStrInf_5 | -16 | -55 | 1 |
| 31 | 17Networks_LH_VisPeri_ExStrSup_1 | -20 | -63 | 7 |
| 32 | 17Networks_LH_VisPeri_ExStrSup_2 | -7 | -68 | 16 |
| 33 | 17Networks_LH_VisPeri_ExStrSup_3 | -17 | -66 | 17 |
| 34 | 17Networks_LH_VisPeri_ExStrSup_4 | -3 | -84 | 25 |
| 35 | 17Networks_LH_VisPeri_ExStrSup_5 | -13 | -75 | 25 |
| 36 | 17Networks_LH_VisPeri_ExStrSup_6 | -14 | -90 | 34 |
| 37 | 17Networks_LH_VisPeri_ExStrSup_7 | -12 | -82 | 37 |
| 38 | 17Networks_LH_SomMotA_1 | -55 | -21 | 47 |
| 39 | 17Networks_LH_SomMotA_2 | -9 | -8 | 42 |
| 40 | 17Networks_LH_SomMotA_3 | -48 | -17 | 52 |
| 41 | 17Networks_LH_SomMotA_4 | -51 | -26 | 55 |
| 42 | 17Networks_LH_SomMotA_5 | -42 | -13 | 49 |
| 43 | 17Networks_LH_SomMotA_6 | -7 | -14 | 49 |
| 44 | 17Networks_LH_SomMotA_7 | -45 | -24 | 63 |
| 45 | 17Networks_LH_SomMotA_8 | -40 | -24 | 53 |
| 46 | 17Networks_LH_SomMotA_9 | -40 | -36 | 63 |
| 47 | 17Networks_LH_SomMotA_10 | -37 | -19 | 63 |
| 48 | 17Networks_LH_SomMotA_11 | -4 | -9 | 61 |
| 49 | 17Networks_LH_SomMotA_12 | -8 | -40 | 58 |
| 50 | 17Networks_LH_SomMotA_13 | -4 | -26 | 57 |
| 51 | 17Networks_LH_SomMotA_14 | -30 | -11 | 63 |
| 52 | 17Networks_LH_SomMotA_15 | -27 | -40 | 59 |
| 53 | 17Networks_LH_SomMotA_16 | -31 | -29 | 62 |
| 54 | 17Networks_LH_SomMotA_17 | -27 | -48 | 65 |
| 55 | 17Networks_LH_SomMotA_18 | -30 | -35 | 70 |
| 56 | 17Networks_LH_SomMotA_19 | -21 | -12 | 71 |
| 57 | 17Networks_LH_SomMotA_20 | -19 | -24 | 68 |
| 58 | 17Networks_LH_SomMotA_21 | -9 | -43 | 71 |
| 59 | 17Networks_LH_SomMotA_22 | -23 | -38 | 71 |
| 60 | 17Networks_LH_SomMotA_23 | -20 | -31 | 67 |
| 61 | 17Networks_LH_SomMotA_24 | -3 | -31 | 68 |
| 62 | 17Networks_LH_SomMotA_25 | -6 | -17 | 73 |
| 63 | 17Networks_LH_SomMotA_26 | -17 | -46 | 72 |
| 64 | 17Networks_LH_SomMotA_27 | -14 | -13 | 73 |
| 65 | 17Networks_LH_SomMotA_28 | -11 | -27 | 73 |
| 66 | 17Networks_LH_SomMotA_29 | -11 | -34 | 74 |
| 67 | 17Networks_LH_SomMotB_Cent_1 | -59 | -3 | 20 |
| 68 | 17Networks_LH_SomMotB_Cent_2 | -64 | -10 | 26 |
| 69 | 17Networks_LH_SomMotB_Cent_3 | -55 | -9 | 31 |
| 70 | 17Networks_LH_SomMotB_Cent_4 | -54 | -4 | 34 |
| 71 | 17Networks_LH_SomMotB_Cent_5 | -58 | -15 | 40 |
| 72 | 17Networks_LH_SomMotB_Cent_6 | -50 | -16 | 41 |
| 73 | 17Networks_LH_SomMotB_Cent_7 | -52 | -4 | 47 |
| 74 | 17Networks_LH_SomMotB_Cent_8 | -42 | -17 | 39 |
| 75 | 17Networks_LH_SomMotB_S2_1 | -35 | -12 | 14 |
| 76 | 17Networks_LH_SomMotB_S2_2 | -40 | -13 | 18 |
| 77 | 17Networks_LH_SomMotB_S2_3 | -38 | -26 | 20 |
| 78 | 17Networks_LH_SomMotB_S2_4 | -51 | -13 | 14 |
| 79 | 17Networks_LH_SomMotB_S2_5 | -59 | 1 | 13 |
| 80 | 17Networks_LH_SomMotB_S2_6 | -62 | -8 | 11 |
| 81 | 17Networks_LH_SomMotB_S2_7 | -48 | -25 | 18 |
| 82 | 17Networks_LH_SomMotB_S2_8 | -61 | -18 | 20 |
| 83 | 17Networks_LH_SomMotB_Ins_1 | -35 | -25 | 13 |
| 84 | 17Networks_LH_SomMotB_Aud_1 | -53 | 0 | -3 |
| 85 | 17Networks_LH_SomMotB_Aud_2 | -58 | -15 | 6 |
| 86 | 17Networks_LH_SomMotB_Aud_3 | -43 | -19 | 4 |
| 87 | 17Networks_LH_SomMotB_Aud_4 | -61 | -25 | 10 |
| 88 | 17Networks_LH_SomMotB_Aud_5 | -47 | -25 | 8 |
| 89 | 17Networks_LH_SomMotB_Aud_6 | -59 | -37 | 15 |
| 90 | 17Networks_LH_SomMotB_Aud_7 | -41 | -35 | 14 |
| 91 | 17Networks_LH_DorsAttnA_TempOcc_1 | -46 | -45 | -20 |
| 92 | 17Networks_LH_DorsAttnA_TempOcc_2 | -25 | -45 | -15 |
| 93 | 17Networks_LH_DorsAttnA_TempOcc_3 | -45 | -62 | -14 |
| 94 | 17Networks_LH_DorsAttnA_TempOcc_4 | -46 | -71 | -7 |
| 95 | 17Networks_LH_DorsAttnA_TempOcc_5 | -48 | -64 | -2 |
| 96 | 17Networks_LH_DorsAttnA_TempOcc_6 | -51 | -65 | 7 |
| 97 | 17Networks_LH_DorsAttnA_ParOcc_1 | -49 | -73 | 13 |
| 98 | 17Networks_LH_DorsAttnA_ParOcc_2 | -43 | -79 | 15 |
| 99 | 17Networks_LH_DorsAttnA_ParOcc_3 | -46 | -62 | 14 |
| 100 | 17Networks_LH_DorsAttnA_ParOcc_4 | -31 | -84 | 29 |
| 101 | 17Networks_LH_DorsAttnA_SPL_1 | -28 | -71 | 30 |
| 102 | 17Networks_LH_DorsAttnA_SPL_2 | -22 | -84 | 43 |
| 103 | 17Networks_LH_DorsAttnA_SPL_3 | -22 | -75 | 37 |
| 104 | 17Networks_LH_DorsAttnA_SPL_4 | -25 | -65 | 43 |
| 105 | 17Networks_LH_DorsAttnA_SPL_5 | -19 | -68 | 47 |
| 106 | 17Networks_LH_DorsAttnA_SPL_6 | -15 | -75 | 51 |
| 107 | 17Networks_LH_DorsAttnA_SPL_7 | -28 | -57 | 54 |
| 108 | 17Networks_LH_DorsAttnA_SPL_8 | -26 | -65 | 56 |
| 109 | 17Networks_LH_DorsAttnA_SPL_9 | -35 | -51 | 54 |
| 110 | 17Networks_LH_DorsAttnA_SPL_10 | -17 | -70 | 59 |
| 111 | 17Networks_LH_DorsAttnA_SPL_11 | -30 | -60 | 64 |
| 112 | 17Networks_LH_DorsAttnB_PostC_1 | -63 | -26 | 36 |
| 113 | 17Networks_LH_DorsAttnB_PostC_2 | -54 | -24 | 39 |
| 114 | 17Networks_LH_DorsAttnB_PostC_3 | -54 | -33 | 45 |
| 115 | 17Networks_LH_DorsAttnB_PostC_4 | -48 | -28 | 44 |
| 116 | 17Networks_LH_DorsAttnB_PostC_5 | -41 | -34 | 46 |
| 117 | 17Networks_LH_DorsAttnB_PostC_6 | -37 | -41 | 55 |
| 118 | 17Networks_LH_DorsAttnB_PostC_7 | -35 | -46 | 62 |
| 119 | 17Networks_LH_DorsAttnB_PostC_8 | -7 | -59 | 62 |
| 120 | 17Networks_LH_DorsAttnB_PostC_9 | -20 | -58 | 66 |
| 121 | 17Networks_LH_DorsAttnB_PostC_10 | -9 | -54 | 71 |
| 122 | 17Networks_LH_DorsAttnB_FEF_1 | -38 | -3 | 51 |
| 123 | 17Networks_LH_DorsAttnB_FEF_2 | -25 | -6 | 53 |
| 124 | 17Networks_LH_DorsAttnB_FEF_3 | -19 | -10 | 62 |
| 125 | 17Networks_LH_DorsAttnB_FEF_4 | -19 | -1 | 69 |
| 126 | 17Networks_LH_DorsAttnB_PrCv_1 | -56 | 7 | 27 |
| 127 | 17Networks_LH_SalVentAttnA_ParOper_1 | -47 | -38 | 24 |
| 128 | 17Networks_LH_SalVentAttnA_ParOper_2 | -58 | -44 | 27 |
| 129 | 17Networks_LH_SalVentAttnA_ParOper_3 | -59 | -29 | 22 |
| 130 | 17Networks_LH_SalVentAttnA_ParOper_4 | -58 | -19 | 30 |
| 131 | 17Networks_LH_SalVentAttnA_ParOper_5 | -61 | -37 | 34 |
| 132 | 17Networks_LH_SalVentAttnA_FrOper_1 | -38 | 3 | 12 |
| 133 | 17Networks_LH_SalVentAttnA_FrOper_2 | -43 | -4 | 13 |
| 134 | 17Networks_LH_SalVentAttnA_FrOper_3 | -51 | 1 | 5 |
| 135 | 17Networks_LH_SalVentAttnA_FrOper_4 | -56 | 8 | 13 |
| 136 | 17Networks_LH_SalVentAttnA_Ins_1 | -39 | 2 | -11 |
| 137 | 17Networks_LH_SalVentAttnA_Ins_2 | -39 | -9 | -9 |
| 138 | 17Networks_LH_SalVentAttnA_Ins_3 | -39 | 2 | 0 |
| 139 | 17Networks_LH_SalVentAttnA_Ins_4 | -40 | -15 | 0 |
| 140 | 17Networks_LH_SalVentAttnA_Ins_5 | -33 | 19 | 8 |
| 141 | 17Networks_LH_SalVentAttnA_Ins_6 | -37 | -3 | 9 |
| 142 | 17Networks_LH_SalVentAttnA_ParMed_1 | -8 | -22 | 39 |
| 143 | 17Networks_LH_SalVentAttnA_ParMed_2 | -12 | -35 | 42 |
| 144 | 17Networks_LH_SalVentAttnA_ParMed_3 | -13 | -24 | 42 |
| 145 | 17Networks_LH_SalVentAttnA_ParMed_4 | -12 | -44 | 51 |
| 146 | 17Networks_LH_SalVentAttnA_ParMed_5 | -11 | -34 | 49 |
| 147 | 17Networks_LH_SalVentAttnA_ParMed_6 | -8 | -48 | 61 |
| 148 | 17Networks_LH_SalVentAttnA_FrMed_1 | -6 | 16 | 36 |
| 149 | 17Networks_LH_SalVentAttnA_FrMed_2 | -5 | 3 | 37 |
| 150 | 17Networks_LH_SalVentAttnA_FrMed_3 | -6 | 4 | 48 |
| 151 | 17Networks_LH_SalVentAttnA_FrMed_4 | -5 | 3 | 59 |
| 152 | 17Networks_LH_SalVentAttnA_FrMed_5 | -8 | -2 | 71 |
| 153 | 17Networks_LH_SalVentAttnB_PFCd_1 | -14 | 9 | 67 |
| 154 | 17Networks_LH_SalVentAttnB_PFCl_1 | -40 | 41 | 21 |
| 155 | 17Networks_LH_SalVentAttnB_PFCl_2 | -30 | 46 | 27 |
| 156 | 17Networks_LH_SalVentAttnB_PFCl_3 | -26 | 37 | 37 |
| 157 | 17Networks_LH_SalVentAttnB_PFCl_4 | -35 | 32 | 38 |
| 158 | 17Networks_LH_SalVentAttnB_Ins_1 | -35 | 14 | -8 |
| 159 | 17Networks_LH_SalVentAttnB_Ins_2 | -32 | 22 | -1 |
| 160 | 17Networks_LH_SalVentAttnB_Ins_3 | -34 | 30 | 0 |
| 161 | 17Networks_LH_SalVentAttnB_Ins_4 | -44 | 11 | 2 |
| 162 | 17Networks_LH_SalVentAttnB_PFCmp_1 | -4 | 15 | 25 |
| 163 | 17Networks_LH_SalVentAttnB_PFCmp_2 | -7 | 27 | 29 |
| 164 | 17Networks_LH_SalVentAttnB_PFCmp_3 | -5 | 14 | 52 |
| 165 | 17Networks_LH_LimbicB_OFC_1 | -23 | 14 | -23 |
| 166 | 17Networks_LH_LimbicB_OFC_2 | -13 | 25 | -21 |
| 167 | 17Networks_LH_LimbicB_OFC_3 | -23 | 30 | -18 |
| 168 | 17Networks_LH_LimbicB_OFC_4 | -4 | 22 | -20 |
| 169 | 17Networks_LH_LimbicB_OFC_5 | -10 | 50 | -22 |
| 170 | 17Networks_LH_LimbicB_OFC_6 | -3 | 42 | -22 |
| 171 | 17Networks_LH_LimbicB_OFC_7 | -25 | 48 | -15 |
| 172 | 17Networks_LH_LimbicB_OFC_8 | -5 | 25 | -11 |
| 173 | 17Networks_LH_LimbicB_OFC_9 | -10 | 64 | -15 |
| 174 | 17Networks_LH_LimbicB_OFC_10 | -20 | 63 | -7 |
| 175 | 17Networks_LH_LimbicA_TempPole_1 | -31 | 0 | -43 |
| 176 | 17Networks_LH_LimbicA_TempPole_2 | -49 | -11 | -38 |
| 177 | 17Networks_LH_LimbicA_TempPole_3 | -25 | -7 | -34 |
| 178 | 17Networks_LH_LimbicA_TempPole_4 | -45 | 5 | -39 |
| 179 | 17Networks_LH_LimbicA_TempPole_5 | -39 | -16 | -31 |
| 180 | 17Networks_LH_LimbicA_TempPole_6 | -25 | 8 | -32 |
| 181 | 17Networks_LH_LimbicA_TempPole_7 | -30 | -16 | -30 |
| 182 | 17Networks_LH_LimbicA_TempPole_8 | -54 | -31 | -27 |
| 183 | 17Networks_LH_LimbicA_TempPole_9 | -39 | 17 | -32 |
| 184 | 17Networks_LH_LimbicA_TempPole_10 | -37 | -36 | -23 |
| 185 | 17Networks_LH_LimbicA_TempPole_11 | -36 | 8 | -25 |
| 186 | 17Networks_LH_LimbicA_TempPole_12 | -45 | 6 | -16 |
| 187 | 17Networks_LH_ContA_Temp_1 | -54 | -55 | -18 |
| 188 | 17Networks_LH_ContA_Temp_2 | -58 | -59 | -2 |
| 189 | 17Networks_LH_ContA_IPS_1 | -29 | -75 | 44 |
| 190 | 17Networks_LH_ContA_IPS_2 | -59 | -42 | 45 |
| 191 | 17Networks_LH_ContA_IPS_3 | -45 | -42 | 47 |
| 192 | 17Networks_LH_ContA_IPS_4 | -31 | -60 | 46 |
| 193 | 17Networks_LH_ContA_IPS_5 | -34 | -50 | 40 |
| 194 | 17Networks_LH_ContA_IPS_6 | -34 | -43 | 41 |
| 195 | 17Networks_LH_ContA_PFCd_1 | -30 | 3 | 59 |
| 196 | 17Networks_LH_ContA_PFCd_2 | -22 | 8 | 60 |
| 197 | 17Networks_LH_ContA_PFCl_1 | -49 | 10 | 14 |
| 198 | 17Networks_LH_ContA_PFCl_2 | -48 | 18 | 24 |
| 199 | 17Networks_LH_ContA_PFCl_3 | -44 | 6 | 25 |
| 200 | 17Networks_LH_ContA_PFCl_4 | -44 | 25 | 27 |
| 201 | 17Networks_LH_ContA_PFCl_5 | -37 | 9 | 32 |
| 202 | 17Networks_LH_ContA_PFCl_6 | -46 | 2 | 37 |
| 203 | 17Networks_LH_ContA_PFClv_1 | -39 | 49 | 7 |
| 204 | 17Networks_LH_ContA_PFClv_2 | -46 | 35 | 13 |
| 205 | 17Networks_LH_ContA_Cingm_1 | -4 | 1 | 30 |
| 206 | 17Networks_LH_ContB_Temp_1 | -61 | -36 | -16 |
| 207 | 17Networks_LH_ContB_Temp_2 | -61 | -48 | -11 |
| 208 | 17Networks_LH_ContB_IPL_1 | -56 | -51 | 38 |
| 209 | 17Networks_LH_ContB_IPL_2 | -51 | -53 | 49 |
| 210 | 17Networks_LH_ContB_IPL_3 | -53 | -46 | 53 |
| 211 | 17Networks_LH_ContB_IPL_4 | -37 | -62 | 51 |
| 212 | 17Networks_LH_ContB_IPL_5 | -43 | -53 | 49 |
| 213 | 17Networks_LH_ContB_PFCd_1 | -29 | 16 | 55 |
| 214 | 17Networks_LH_ContB_PFCl_1 | -45 | 20 | 36 |
| 215 | 17Networks_LH_ContB_PFCl_2 | -45 | 12 | 40 |
| 216 | 17Networks_LH_ContB_PFClv_1 | -41 | 50 | -7 |
| 217 | 17Networks_LH_ContB_PFClv_2 | -29 | 58 | -1 |
| 218 | 17Networks_LH_ContB_PFClv_3 | -30 | 56 | 11 |
| 219 | 17Networks_LH_ContB_PFCmp_1 | -4 | 27 | 46 |
| 220 | 17Networks_LH_ContC_pCun_1 | -10 | -70 | 31 |
| 221 | 17Networks_LH_ContC_pCun_2 | -9 | -76 | 43 |
| 222 | 17Networks_LH_ContC_pCun_3 | -3 | -68 | 42 |
| 223 | 17Networks_LH_ContC_pCun_4 | -5 | -71 | 54 |
| 224 | 17Networks_LH_ContC_pCun_5 | -6 | -62 | 51 |
| 225 | 17Networks_LH_ContC_pCun_6 | -4 | -51 | 53 |
| 226 | 17Networks_LH_ContC_Cingp_1 | -6 | -40 | 24 |
| 227 | 17Networks_LH_ContC_Cingp_2 | -4 | -22 | 29 |
| 228 | 17Networks_LH_DefaultA_IPL_1 | -45 | -63 | 24 |
| 229 | 17Networks_LH_DefaultA_IPL_2 | -48 | -68 | 34 |
| 230 | 17Networks_LH_DefaultA_IPL_3 | -39 | -74 | 44 |
| 231 | 17Networks_LH_DefaultA_IPL_4 | -45 | -64 | 49 |
| 232 | 17Networks_LH_DefaultA_PFCd_1 | -20 | 40 | 42 |
| 233 | 17Networks_LH_DefaultA_PFCd_2 | -25 | 27 | 44 |
| 234 | 17Networks_LH_DefaultA_PFCd_3 | -22 | 24 | 52 |
| 235 | 17Networks_LH_DefaultA_pCunPCC_1 | -6 | -47 | 15 |
| 236 | 17Networks_LH_DefaultA_pCunPCC_2 | -4 | -53 | 19 |
| 237 | 17Networks_LH_DefaultA_pCunPCC_3 | -4 | -66 | 24 |
| 238 | 17Networks_LH_DefaultA_pCunPCC_4 | -7 | -56 | 27 |
| 239 | 17Networks_LH_DefaultA_pCunPCC_5 | -4 | -43 | 30 |
| 240 | 17Networks_LH_DefaultA_pCunPCC_6 | -5 | -62 | 33 |
| 241 | 17Networks_LH_DefaultA_pCunPCC_7 | -4 | -31 | 36 |
| 242 | 17Networks_LH_DefaultA_pCunPCC_8 | -2 | -15 | 37 |
| 243 | 17Networks_LH_DefaultA_pCunPCC_9 | -12 | -49 | 35 |
| 244 | 17Networks_LH_DefaultA_pCunPCC_10 | -5 | -39 | 40 |
| 245 | 17Networks_LH_DefaultA_pCunPCC_11 | -5 | -57 | 43 |
| 246 | 17Networks_LH_DefaultA_pCunPCC_12 | -7 | -48 | 43 |
| 247 | 17Networks_LH_DefaultA_PFCm_1 | -5 | 55 | -10 |
| 248 | 17Networks_LH_DefaultA_PFCm_2 | -6 | 39 | -7 |
| 249 | 17Networks_LH_DefaultA_PFCm_3 | -12 | 69 | 5 |
| 250 | 17Networks_LH_DefaultA_PFCm_4 | -6 | 45 | 6 |
| 251 | 17Networks_LH_DefaultA_PFCm_5 | -22 | 63 | 11 |
| 252 | 17Networks_LH_DefaultA_PFCm_6 | -6 | 57 | 15 |
| 253 | 17Networks_LH_DefaultA_PFCm_7 | -3 | 30 | 16 |
| 254 | 17Networks_LH_DefaultA_PFCm_8 | -7 | 36 | 22 |
| 255 | 17Networks_LH_DefaultB_Temp_1 | -55 | 0 | -29 |
| 256 | 17Networks_LH_DefaultB_Temp_2 | -60 | -19 | -23 |
| 257 | 17Networks_LH_DefaultB_Temp_3 | -52 | 8 | -19 |
| 258 | 17Networks_LH_DefaultB_Temp_4 | -61 | -10 | -15 |
| 259 | 17Networks_LH_DefaultB_Temp_5 | -63 | -33 | -4 |
| 260 | 17Networks_LH_DefaultB_Temp_6 | -51 | -22 | -8 |
| 261 | 17Networks_LH_DefaultB_Temp_7 | -51 | -34 | 0 |
| 262 | 17Networks_LH_DefaultB_IPL_1 | -45 | -56 | 19 |
| 263 | 17Networks_LH_DefaultB_IPL_2 | -49 | -54 | 26 |
| 264 | 17Networks_LH_DefaultB_IPL_3 | -61 | -53 | 28 |
| 265 | 17Networks_LH_DefaultB_IPL_4 | -49 | -59 | 38 |
| 266 | 17Networks_LH_DefaultB_PFCd_1 | -14 | 62 | 24 |
| 267 | 17Networks_LH_DefaultB_PFCd_2 | -22 | 54 | 27 |
| 268 | 17Networks_LH_DefaultB_PFCd_3 | -4 | 47 | 35 |
| 269 | 17Networks_LH_DefaultB_PFCd_4 | -13 | 47 | 44 |
| 270 | 17Networks_LH_DefaultB_PFCd_5 | -6 | 37 | 54 |
| 271 | 17Networks_LH_DefaultB_PFCd_6 | -14 | 23 | 61 |
| 272 | 17Networks_LH_DefaultB_PFCd_7 | -5 | 13 | 64 |
| 273 | 17Networks_LH_DefaultB_PFCl_1 | -39 | 19 | 50 |
| 274 | 17Networks_LH_DefaultB_PFCl_2 | -42 | 7 | 49 |
| 275 | 17Networks_LH_DefaultB_PFCv_1 | -28 | 17 | -16 |
| 276 | 17Networks_LH_DefaultB_PFCv_2 | -36 | 37 | -13 |
| 277 | 17Networks_LH_DefaultB_PFCv_3 | -37 | 24 | -15 |
| 278 | 17Networks_LH_DefaultB_PFCv_4 | -47 | 32 | -10 |
| 279 | 17Networks_LH_DefaultB_PFCv_5 | -48 | 27 | 1 |
| 280 | 17Networks_LH_DefaultB_PFCv_6 | -49 | 40 | -1 |
| 281 | 17Networks_LH_DefaultB_PFCv_7 | -53 | 19 | 10 |
| 282 | 17Networks_LH_DefaultC_IPL_1 | -43 | -72 | 24 |
| 283 | 17Networks_LH_DefaultC_IPL_2 | -39 | -81 | 33 |
| 284 | 17Networks_LH_DefaultC_Rsp_1 | -12 | -46 | 3 |
| 285 | 17Networks_LH_DefaultC_Rsp_2 | -10 | -53 | 8 |
| 286 | 17Networks_LH_DefaultC_Rsp_3 | -12 | -59 | 17 |
| 287 | 17Networks_LH_DefaultC_Rsp_4 | -15 | -65 | 24 |
| 288 | 17Networks_LH_DefaultC_PHC_1 | -21 | -19 | -27 |
| 289 | 17Networks_LH_DefaultC_PHC_2 | -31 | -28 | -22 |
| 290 | 17Networks_LH_DefaultC_PHC_3 | -31 | -41 | -10 |
| 291 | 17Networks_LH_DefaultC_PHC_4 | -21 | -35 | -15 |
| 292 | 17Networks_LH_DefaultC_PHC_5 | -15 | -37 | -11 |
| 293 | 17Networks_LH_TempPar_1 | -53 | -4 | -11 |
| 294 | 17Networks_LH_TempPar_2 | -60 | -18 | -2 |
| 295 | 17Networks_LH_TempPar_3 | -63 | -50 | 4 |
| 296 | 17Networks_LH_TempPar_4 | -52 | -44 | 6 |
| 297 | 17Networks_LH_TempPar_5 | -63 | -35 | 7 |
| 298 | 17Networks_LH_TempPar_6 | -56 | -56 | 13 |
| 299 | 17Networks_LH_TempPar_7 | -47 | -49 | 14 |
| 300 | 17Networks_LH_TempPar_8 | -60 | -49 | 16 |
| 301 | 17Networks_RH_VisCent_Striate_1 | 9 | -92 | -11 |
| 302 | 17Networks_RH_VisCent_Striate_2 | 8 | -89 | 3 |
| 303 | 17Networks_RH_VisCent_Striate_3 | 12 | -99 | 2 |
| 304 | 17Networks_RH_VisCent_ExStr_1 | 36 | -51 | -18 |
| 305 | 17Networks_RH_VisCent_ExStr_2 | 29 | -50 | -12 |
| 306 | 17Networks_RH_VisCent_ExStr_3 | 37 | -73 | -15 |
| 307 | 17Networks_RH_VisCent_ExStr_4 | 26 | -75 | -11 |
| 308 | 17Networks_RH_VisCent_ExStr_5 | 22 | -66 | -8 |
| 309 | 17Networks_RH_VisCent_ExStr_6 | 20 | -88 | -12 |
| 310 | 17Networks_RH_VisCent_ExStr_7 | 41 | -84 | -16 |
| 311 | 17Networks_RH_VisCent_ExStr_8 | 12 | -80 | -13 |
| 312 | 17Networks_RH_VisCent_ExStr_9 | 28 | -95 | -10 |
| 313 | 17Networks_RH_VisCent_ExStr_10 | 43 | -85 | -5 |
| 314 | 17Networks_RH_VisCent_ExStr_11 | 23 | -100 | 2 |
| 315 | 17Networks_RH_VisCent_ExStr_12 | 33 | -91 | 4 |
| 316 | 17Networks_RH_VisCent_ExStr_13 | 23 | -97 | 14 |
| 317 | 17Networks_RH_VisCent_ExStr_14 | 43 | -79 | 10 |
| 318 | 17Networks_RH_VisCent_ExStr_15 | 12 | -94 | 19 |
| 319 | 17Networks_RH_VisCent_ExStr_16 | 29 | -88 | 17 |
| 320 | 17Networks_RH_VisCent_ExStr_17 | 22 | -87 | 30 |
| 321 | 17Networks_RH_VisCent_ExStr_18 | 30 | -77 | 25 |
| 322 | 17Networks_RH_VisPeri_StriCal_1 | 4 | -81 | -3 |
| 323 | 17Networks_RH_VisPeri_StriCal_2 | 11 | -60 | 5 |
| 324 | 17Networks_RH_VisPeri_StriCal_3 | 10 | -73 | 9 |
| 325 | 17Networks_RH_VisPeri_StriCal_4 | 23 | -59 | 7 |
| 326 | 17Networks_RH_VisPeri_ExStrInf_1 | 24 | -46 | -8 |
| 327 | 17Networks_RH_VisPeri_ExStrInf_2 | 14 | -58 | -5 |
| 328 | 17Networks_RH_VisPeri_ExStrInf_3 | 9 | -71 | -4 |
| 329 | 17Networks_RH_VisPeri_ExStrInf_4 | 15 | -39 | -6 |
| 330 | 17Networks_RH_VisPeri_ExStrInf_5 | 18 | -50 | 0 |
| 331 | 17Networks_RH_VisPeri_ExStrSup_1 | 5 | -85 | 12 |
| 332 | 17Networks_RH_VisPeri_ExStrSup_2 | 16 | -66 | 19 |
| 333 | 17Networks_RH_VisPeri_ExStrSup_3 | 5 | -79 | 24 |
| 334 | 17Networks_RH_VisPeri_ExStrSup_4 | 8 | -87 | 34 |
| 335 | 17Networks_RH_VisPeri_ExStrSup_5 | 14 | -77 | 31 |
| 336 | 17Networks_RH_VisPeri_ExStrSup_6 | 18 | -83 | 42 |
| 337 | 17Networks_RH_SomMotA_1 | 53 | -19 | 47 |
| 338 | 17Networks_RH_SomMotA_2 | 51 | -13 | 50 |
| 339 | 17Networks_RH_SomMotA_3 | 46 | -9 | 51 |
| 340 | 17Networks_RH_SomMotA_4 | 49 | -26 | 57 |
| 341 | 17Networks_RH_SomMotA_5 | 7 | -13 | 50 |
| 342 | 17Networks_RH_SomMotA_6 | 43 | -21 | 54 |
| 343 | 17Networks_RH_SomMotA_7 | 41 | -17 | 57 |
| 344 | 17Networks_RH_SomMotA_8 | 34 | -34 | 60 |
| 345 | 17Networks_RH_SomMotA_9 | 5 | -23 | 57 |
| 346 | 17Networks_RH_SomMotA_10 | 35 | -27 | 61 |
| 347 | 17Networks_RH_SomMotA_11 | 36 | -16 | 68 |
| 348 | 17Networks_RH_SomMotA_12 | 30 | -41 | 65 |
| 349 | 17Networks_RH_SomMotA_13 | 31 | -23 | 61 |
| 350 | 17Networks_RH_SomMotA_14 | 28 | -11 | 65 |
| 351 | 17Networks_RH_SomMotA_15 | 9 | -41 | 65 |
| 352 | 17Networks_RH_SomMotA_16 | 3 | -32 | 63 |
| 353 | 17Networks_RH_SomMotA_17 | 25 | -34 | 69 |
| 354 | 17Networks_RH_SomMotA_18 | 24 | -28 | 68 |
| 355 | 17Networks_RH_SomMotA_19 | 19 | -27 | 62 |
| 356 | 17Networks_RH_SomMotA_20 | 23 | -22 | 71 |
| 357 | 17Networks_RH_SomMotA_21 | 19 | -14 | 72 |
| 358 | 17Networks_RH_SomMotA_22 | 7 | -16 | 73 |
| 359 | 17Networks_RH_SomMotA_23 | 21 | -37 | 75 |
| 360 | 17Networks_RH_SomMotA_24 | 5 | -31 | 73 |
| 361 | 17Networks_RH_SomMotA_25 | 11 | -38 | 77 |
| 362 | 17Networks_RH_SomMotA_26 | 14 | -26 | 72 |
| 363 | 17Networks_RH_SomMotA_27 | 14 | -32 | 74 |
| 364 | 17Networks_RH_SomMotB_Cent_1 | 61 | -4 | 24 |
| 365 | 17Networks_RH_SomMotB_Cent_2 | 63 | -12 | 28 |
| 366 | 17Networks_RH_SomMotB_Cent_3 | 61 | 7 | 31 |
| 367 | 17Networks_RH_SomMotB_Cent_4 | 53 | -5 | 38 |
| 368 | 17Networks_RH_SomMotB_Cent_5 | 52 | -11 | 38 |
| 369 | 17Networks_RH_SomMotB_Cent_6 | 39 | -16 | 41 |
| 370 | 17Networks_RH_SomMotB_S2_1 | 37 | -9 | 12 |
| 371 | 17Networks_RH_SomMotB_S2_2 | 35 | -21 | 13 |
| 372 | 17Networks_RH_SomMotB_S2_3 | 41 | -12 | 18 |
| 373 | 17Networks_RH_SomMotB_S2_4 | 41 | -29 | 18 |
| 374 | 17Networks_RH_SomMotB_S2_5 | 50 | -9 | 13 |
| 375 | 17Networks_RH_SomMotB_S2_6 | 60 | 0 | 11 |
| 376 | 17Networks_RH_SomMotB_S2_7 | 49 | -20 | 18 |
| 377 | 17Networks_RH_SomMotB_S2_8 | 61 | -12 | 15 |
| 378 | 17Networks_RH_SomMotB_Ins_1 | 40 | -19 | 4 |
| 379 | 17Networks_RH_SomMotB_Aud_1 | 54 | 4 | -5 |
| 380 | 17Networks_RH_SomMotB_Aud_2 | 56 | -11 | 4 |
| 381 | 17Networks_RH_SomMotB_Aud_3 | 47 | -22 | 10 |
| 382 | 17Networks_RH_SomMotB_Aud_4 | 61 | -24 | 11 |
| 383 | 17Networks_RH_DorsAttnA_TempOcc_1 | 47 | -37 | -22 |
| 384 | 17Networks_RH_DorsAttnA_TempOcc_2 | 50 | -53 | -17 |
| 385 | 17Networks_RH_DorsAttnA_TempOcc_3 | 51 | -65 | -8 |
| 386 | 17Networks_RH_DorsAttnA_ParOcc_1 | 48 | -67 | 4 |
| 387 | 17Networks_RH_DorsAttnA_ParOcc_2 | 51 | -59 | 14 |
| 388 | 17Networks_RH_DorsAttnA_ParOcc_3 | 43 | -81 | 20 |
| 389 | 17Networks_RH_DorsAttnA_ParOcc_4 | 36 | -80 | 25 |
| 390 | 17Networks_RH_DorsAttnA_SPL_1 | 30 | -66 | 34 |
| 391 | 17Networks_RH_DorsAttnA_SPL_2 | 29 | -78 | 41 |
| 392 | 17Networks_RH_DorsAttnA_SPL_3 | 16 | -79 | 52 |
| 393 | 17Networks_RH_DorsAttnA_SPL_4 | 28 | -63 | 52 |
| 394 | 17Networks_RH_DorsAttnA_SPL_5 | 34 | -56 | 52 |
| 395 | 17Networks_RH_DorsAttnA_SPL_6 | 21 | -69 | 54 |
| 396 | 17Networks_RH_DorsAttnA_SPL_7 | 35 | -46 | 54 |
| 397 | 17Networks_RH_DorsAttnA_SPL_8 | 11 | -71 | 54 |
| 398 | 17Networks_RH_DorsAttnA_SPL_9 | 27 | -56 | 60 |
| 399 | 17Networks_RH_DorsAttnA_SPL_10 | 8 | -64 | 59 |
| 400 | 17Networks_RH_DorsAttnA_SPL_11 | 22 | -63 | 65 |
| 401 | 17Networks_RH_DorsAttnA_SPL_12 | 14 | -61 | 66 |
| 402 | 17Networks_RH_DorsAttnB_TempOcc_1 | 59 | -55 | -1 |
| 403 | 17Networks_RH_DorsAttnB_PostC_1 | 62 | -18 | 32 |
| 404 | 17Networks_RH_DorsAttnB_PostC_2 | 63 | -25 | 44 |
| 405 | 17Networks_RH_DorsAttnB_PostC_3 | 58 | -14 | 39 |
| 406 | 17Networks_RH_DorsAttnB_PostC_4 | 56 | -22 | 42 |
| 407 | 17Networks_RH_DorsAttnB_PostC_5 | 46 | -28 | 43 |
| 408 | 17Networks_RH_DorsAttnB_PostC_6 | 39 | -40 | 46 |
| 409 | 17Networks_RH_DorsAttnB_PostC_7 | 36 | -33 | 47 |
| 410 | 17Networks_RH_DorsAttnB_PostC_8 | 36 | -40 | 58 |
| 411 | 17Networks_RH_DorsAttnB_PostC_9 | 36 | -49 | 66 |
| 412 | 17Networks_RH_DorsAttnB_PostC_10 | 8 | -51 | 65 |
| 413 | 17Networks_RH_DorsAttnB_PostC_11 | 23 | -49 | 69 |
| 414 | 17Networks_RH_DorsAttnB_PostC_12 | 15 | -47 | 75 |
| 415 | 17Networks_RH_DorsAttnB_FEF_1 | 40 | -3 | 51 |
| 416 | 17Networks_RH_DorsAttnB_FEF_2 | 30 | -7 | 53 |
| 417 | 17Networks_RH_DorsAttnB_FEF_3 | 25 | -1 | 59 |
| 418 | 17Networks_RH_DorsAttnB_FEF_4 | 19 | -4 | 69 |
| 419 | 17Networks_RH_DorsAttnB_PrCv_1 | 52 | 8 | 30 |
| 420 | 17Networks_RH_SalVentAttnA_ParOper_1 | 64 | -31 | 22 |
| 421 | 17Networks_RH_SalVentAttnA_ParOper_2 | 51 | -30 | 25 |
| 422 | 17Networks_RH_SalVentAttnA_ParOper_3 | 60 | -21 | 21 |
| 423 | 17Networks_RH_SalVentAttnA_ParOper_4 | 59 | -27 | 33 |
| 424 | 17Networks_RH_SalVentAttnA_ParOper_5 | 65 | -30 | 34 |
| 425 | 17Networks_RH_SalVentAttnA_PrC_1 | 50 | 3 | 41 |
| 426 | 17Networks_RH_SalVentAttnA_FrOper_1 | 43 | 13 | 5 |
| 427 | 17Networks_RH_SalVentAttnA_FrOper_2 | 42 | -1 | 14 |
| 428 | 17Networks_RH_SalVentAttnA_FrOper_3 | 50 | 4 | 4 |
| 429 | 17Networks_RH_SalVentAttnA_FrOper_4 | 53 | 11 | 13 |
| 430 | 17Networks_RH_SalVentAttnA_FrOper_5 | 59 | 9 | 19 |
| 431 | 17Networks_RH_SalVentAttnA_Ins_1 | 39 | 7 | -16 |
| 432 | 17Networks_RH_SalVentAttnA_Ins_2 | 46 | 7 | -15 |
| 433 | 17Networks_RH_SalVentAttnA_Ins_3 | 41 | 6 | -5 |
| 434 | 17Networks_RH_SalVentAttnA_Ins_4 | 41 | -10 | -3 |
| 435 | 17Networks_RH_SalVentAttnA_Ins_5 | 38 | 2 | 5 |
| 436 | 17Networks_RH_SalVentAttnA_Ins_6 | 36 | 6 | 12 |
| 437 | 17Networks_RH_SalVentAttnA_ParMed_1 | 10 | -29 | 41 |
| 438 | 17Networks_RH_SalVentAttnA_ParMed_2 | 11 | -16 | 41 |
| 439 | 17Networks_RH_SalVentAttnA_ParMed_3 | 12 | -38 | 45 |
| 440 | 17Networks_RH_SalVentAttnA_ParMed_4 | 13 | -26 | 45 |
| 441 | 17Networks_RH_SalVentAttnA_ParMed_5 | 10 | -43 | 53 |
| 442 | 17Networks_RH_SalVentAttnA_ParMed_6 | 10 | -34 | 52 |
| 443 | 17Networks_RH_SalVentAttnA_FrMed_1 | 7 | 12 | 37 |
| 444 | 17Networks_RH_SalVentAttnA_FrMed_2 | 4 | -8 | 37 |
| 445 | 17Networks_RH_SalVentAttnA_FrMed_3 | 8 | 0 | 44 |
| 446 | 17Networks_RH_SalVentAttnA_FrMed_4 | 8 | 14 | 48 |
| 447 | 17Networks_RH_SalVentAttnA_FrMed_5 | 6 | 0 | 55 |
| 448 | 17Networks_RH_SalVentAttnA_FrMed_6 | 7 | -2 | 67 |
| 449 | 17Networks_RH_SalVentAttnA_FrMed_7 | 16 | 6 | 70 |
| 450 | 17Networks_RH_SalVentAttnB_IPL_1 | 63 | -39 | 30 |
| 451 | 17Networks_RH_SalVentAttnB_IPL_2 | 61 | -35 | 44 |
| 452 | 17Networks_RH_SalVentAttnB_PFCd_1 | 24 | 9 | 64 |
| 453 | 17Networks_RH_SalVentAttnB_PFCd_2 | 14 | 16 | 64 |
| 454 | 17Networks_RH_SalVentAttnB_PFCl_1 | 32 | 48 | 20 |
| 455 | 17Networks_RH_SalVentAttnB_PFCl_2 | 25 | 54 | 26 |
| 456 | 17Networks_RH_SalVentAttnB_PFCl_3 | 33 | 43 | 31 |
| 457 | 17Networks_RH_SalVentAttnB_PFClv_1 | 50 | 40 | 5 |
| 458 | 17Networks_RH_SalVentAttnB_PFClv_2 | 41 | 50 | 7 |
| 459 | 17Networks_RH_SalVentAttnB_Ins_1 | 34 | 20 | -8 |
| 460 | 17Networks_RH_SalVentAttnB_Ins_2 | 34 | 26 | 1 |
| 461 | 17Networks_RH_SalVentAttnB_Ins_3 | 38 | 23 | 7 |
| 462 | 17Networks_RH_SalVentAttnB_Ins_4 | 54 | 15 | 6 |
| 463 | 17Networks_RH_SalVentAttnB_PFCmp_1 | 7 | 35 | 24 |
| 464 | 17Networks_RH_SalVentAttnB_PFCmp_2 | 9 | 25 | 32 |
| 465 | 17Networks_RH_SalVentAttnB_PFCmp_3 | 5 | 25 | 48 |
| 466 | 17Networks_RH_SalVentAttnB_PFCmp_4 | 5 | 12 | 61 |
| 467 | 17Networks_RH_SalVentAttnB_Cinga_1 | 5 | 21 | 24 |
| 468 | 17Networks_RH_LimbicB_OFC_1 | 20 | 14 | -22 |
| 469 | 17Networks_RH_LimbicB_OFC_2 | 15 | 28 | -24 |
| 470 | 17Networks_RH_LimbicB_OFC_3 | 11 | 23 | -18 |
| 471 | 17Networks_RH_LimbicB_OFC_4 | 10 | 42 | -23 |
| 472 | 17Networks_RH_LimbicB_OFC_5 | 23 | 28 | -19 |
| 473 | 17Networks_RH_LimbicB_OFC_6 | 23 | 42 | -17 |
| 474 | 17Networks_RH_LimbicB_OFC_7 | 5 | 21 | -21 |
| 475 | 17Networks_RH_LimbicB_OFC_8 | 4 | 50 | -23 |
| 476 | 17Networks_RH_LimbicB_OFC_9 | 14 | 58 | -19 |
| 477 | 17Networks_RH_LimbicB_OFC_10 | 4 | 27 | -11 |
| 478 | 17Networks_RH_LimbicB_OFC_11 | 6 | 63 | -11 |
| 479 | 17Networks_RH_LimbicA_TempPole_1 | 37 | 0 | -44 |
| 480 | 17Networks_RH_LimbicA_TempPole_2 | 33 | 14 | -40 |
| 481 | 17Networks_RH_LimbicA_TempPole_3 | 28 | -6 | -36 |
| 482 | 17Networks_RH_LimbicA_TempPole_4 | 39 | -12 | -34 |
| 483 | 17Networks_RH_LimbicA_TempPole_5 | 24 | 6 | -37 |
| 484 | 17Networks_RH_LimbicA_TempPole_6 | 52 | -18 | -32 |
| 485 | 17Networks_RH_LimbicA_TempPole_7 | 31 | 14 | -30 |
| 486 | 17Networks_RH_LimbicA_TempPole_8 | 37 | -31 | -24 |
| 487 | 17Networks_RH_LimbicA_TempPole_9 | 44 | 19 | -26 |
| 488 | 17Networks_RH_ContA_IPS_1 | 34 | -72 | 41 |
| 489 | 17Networks_RH_ContA_IPS_2 | 54 | -39 | 48 |
| 490 | 17Networks_RH_ContA_IPS_3 | 55 | -30 | 52 |
| 491 | 17Networks_RH_ContA_IPS_4 | 48 | -37 | 53 |
| 492 | 17Networks_RH_ContA_IPS_5 | 40 | -62 | 49 |
| 493 | 17Networks_RH_ContA_IPS_6 | 44 | -45 | 45 |
| 494 | 17Networks_RH_ContA_IPS_7 | 33 | -66 | 50 |
| 495 | 17Networks_RH_ContA_IPS_8 | 37 | -51 | 42 |
| 496 | 17Networks_RH_ContA_PFCd_1 | 29 | 2 | 53 |
| 497 | 17Networks_RH_ContA_PFCd_2 | 24 | 12 | 51 |
| 498 | 17Networks_RH_ContA_PFCl_1 | 50 | 30 | 18 |
| 499 | 17Networks_RH_ContA_PFCl_2 | 54 | 18 | 20 |
| 500 | 17Networks_RH_ContA_PFCl_3 | 42 | 43 | 16 |
| 501 | 17Networks_RH_ContA_PFCl_4 | 43 | 18 | 27 |
| 502 | 17Networks_RH_ContA_PFCl_5 | 47 | 30 | 28 |
| 503 | 17Networks_RH_ContA_PFCl_6 | 43 | 8 | 25 |
| 504 | 17Networks_RH_ContA_PFCl_7 | 38 | 10 | 35 |
| 505 | 17Networks_RH_ContA_Cingm_1 | 5 | 4 | 29 |
| 506 | 17Networks_RH_ContB_Temp_1 | 59 | -30 | -23 |
| 507 | 17Networks_RH_ContB_Temp_2 | 65 | -24 | -16 |
| 508 | 17Networks_RH_ContB_Temp_3 | 63 | -41 | -11 |
| 509 | 17Networks_RH_ContB_IPL_1 | 58 | -45 | 35 |
| 510 | 17Networks_RH_ContB_IPL_2 | 42 | -71 | 44 |
| 511 | 17Networks_RH_ContB_IPL_3 | 56 | -52 | 43 |
| 512 | 17Networks_RH_ContB_IPL_4 | 47 | -56 | 51 |
| 513 | 17Networks_RH_ContB_IPL_5 | 50 | -45 | 51 |
| 514 | 17Networks_RH_ContB_PFCld_1 | 38 | 33 | 39 |
| 515 | 17Networks_RH_ContB_PFCld_2 | 47 | 18 | 39 |
| 516 | 17Networks_RH_ContB_PFCld_3 | 42 | 12 | 45 |
| 517 | 17Networks_RH_ContB_PFCld_4 | 40 | 23 | 50 |
| 518 | 17Networks_RH_ContB_PFCld_5 | 41 | 7 | 55 |
| 519 | 17Networks_RH_ContB_PFCld_6 | 31 | 16 | 53 |
| 520 | 17Networks_RH_ContB_PFCld_7 | 23 | 25 | 54 |
| 521 | 17Networks_RH_ContB_PFClv_1 | 35 | 37 | -13 |
| 522 | 17Networks_RH_ContB_PFClv_2 | 28 | 56 | -13 |
| 523 | 17Networks_RH_ContB_PFClv_3 | 42 | 51 | -8 |
| 524 | 17Networks_RH_ContB_PFClv_4 | 30 | 58 | -3 |
| 525 | 17Networks_RH_ContB_PFClv_5 | 19 | 65 | -3 |
| 526 | 17Networks_RH_ContB_PFClv_6 | 30 | 57 | 9 |
| 527 | 17Networks_RH_ContB_PFCmp_1 | 5 | 37 | 48 |
| 528 | 17Networks_RH_ContC_pCun_1 | 17 | -64 | 28 |
| 529 | 17Networks_RH_ContC_pCun_2 | 14 | -72 | 40 |
| 530 | 17Networks_RH_ContC_pCun_3 | 8 | -50 | 44 |
| 531 | 17Networks_RH_ContC_pCun_4 | 7 | -61 | 45 |
| 532 | 17Networks_RH_ContC_pCun_5 | 5 | -71 | 48 |
| 533 | 17Networks_RH_ContC_pCun_6 | 6 | -54 | 53 |
| 534 | 17Networks_RH_ContC_Cingp_1 | 7 | -38 | 25 |
| 535 | 17Networks_RH_ContC_Cingp_2 | 6 | -23 | 29 |
| 536 | 17Networks_RH_ContC_Cingp_3 | 5 | -10 | 31 |
| 537 | 17Networks_RH_DefaultA_Temp_1 | 60 | -5 | -16 |
| 538 | 17Networks_RH_DefaultA_IPL_1 | 50 | -50 | 18 |
| 539 | 17Networks_RH_DefaultA_IPL_2 | 47 | -59 | 25 |
| 540 | 17Networks_RH_DefaultA_IPL_3 | 55 | -53 | 28 |
| 541 | 17Networks_RH_DefaultA_IPL_4 | 50 | -62 | 37 |
| 542 | 17Networks_RH_DefaultA_PFCd_1 | 24 | 37 | 34 |
| 543 | 17Networks_RH_DefaultA_PFCd_2 | 22 | 41 | 45 |
| 544 | 17Networks_RH_DefaultA_PFCd_3 | 29 | 29 | 41 |
| 545 | 17Networks_RH_DefaultA_pCunPCC_1 | 7 | -46 | 15 |
| 546 | 17Networks_RH_DefaultA_pCunPCC_2 | 5 | -53 | 20 |
| 547 | 17Networks_RH_DefaultA_pCunPCC_3 | 4 | -45 | 29 |
| 548 | 17Networks_RH_DefaultA_pCunPCC_4 | 5 | -63 | 28 |
| 549 | 17Networks_RH_DefaultA_pCunPCC_5 | 11 | -49 | 29 |
| 550 | 17Networks_RH_DefaultA_pCunPCC_6 | 6 | -36 | 34 |
| 551 | 17Networks_RH_DefaultA_pCunPCC_7 | 4 | -24 | 37 |
| 552 | 17Networks_RH_DefaultA_pCunPCC_8 | 5 | -67 | 36 |
| 553 | 17Networks_RH_DefaultA_pCunPCC_9 | 9 | -54 | 35 |
| 554 | 17Networks_RH_DefaultA_pCunPCC_10 | 10 | -43 | 38 |
| 555 | 17Networks_RH_DefaultA_PFCm_1 | 6 | 46 | -11 |
| 556 | 17Networks_RH_DefaultA_PFCm_2 | 8 | 53 | -2 |
| 557 | 17Networks_RH_DefaultA_PFCm_3 | 6 | 39 | -1 |
| 558 | 17Networks_RH_DefaultA_PFCm_4 | 8 | 67 | 3 |
| 559 | 17Networks_RH_DefaultA_PFCm_5 | 7 | 56 | 11 |
| 560 | 17Networks_RH_DefaultA_PFCm_6 | 9 | 43 | 10 |
| 561 | 17Networks_RH_DefaultA_PFCm_7 | 18 | 65 | 15 |
| 562 | 17Networks_RH_DefaultA_PFCm_8 | 6 | 30 | 13 |
| 563 | 17Networks_RH_DefaultB_Temp_1 | 63 | -24 | -7 |
| 564 | 17Networks_RH_DefaultB_Temp_2 | 63 | -37 | 0 |
| 565 | 17Networks_RH_DefaultB_AntTemp_1 | 48 | 10 | -35 |
| 566 | 17Networks_RH_DefaultB_AntTemp_2 | 58 | -8 | -27 |
| 567 | 17Networks_RH_DefaultB_AntTemp_3 | 52 | 8 | -22 |
| 568 | 17Networks_RH_DefaultB_PFCd_1 | 7 | 61 | 27 |
| 569 | 17Networks_RH_DefaultB_PFCd_2 | 5 | 48 | 30 |
| 570 | 17Networks_RH_DefaultB_PFCd_3 | 16 | 54 | 34 |
| 571 | 17Networks_RH_DefaultB_PFCd_4 | 10 | 50 | 46 |
| 572 | 17Networks_RH_DefaultB_PFCd_5 | 15 | 37 | 53 |
| 573 | 17Networks_RH_DefaultB_PFCd_6 | 9 | 26 | 60 |
| 574 | 17Networks_RH_DefaultB_PFCv_1 | 29 | 19 | -20 |
| 575 | 17Networks_RH_DefaultB_PFCv_2 | 39 | 25 | -16 |
| 576 | 17Networks_RH_DefaultB_PFCv_3 | 47 | 31 | -11 |
| 577 | 17Networks_RH_DefaultB_PFCv_4 | 50 | 32 | -2 |
| 578 | 17Networks_RH_DefaultB_PFCv_5 | 54 | 23 | 7 |
| 579 | 17Networks_RH_DefaultC_IPL_1 | 47 | -67 | 20 |
| 580 | 17Networks_RH_DefaultC_IPL_2 | 45 | -77 | 27 |
| 581 | 17Networks_RH_DefaultC_IPL_3 | 50 | -69 | 31 |
| 582 | 17Networks_RH_DefaultC_Rsp_1 | 13 | -45 | 3 |
| 583 | 17Networks_RH_DefaultC_Rsp_2 | 10 | -51 | 9 |
| 584 | 17Networks_RH_DefaultC_Rsp_3 | 15 | -57 | 20 |
| 585 | 17Networks_RH_DefaultC_PHC_1 | 22 | -17 | -27 |
| 586 | 17Networks_RH_DefaultC_PHC_2 | 30 | -30 | -19 |
| 587 | 17Networks_RH_DefaultC_PHC_3 | 18 | -31 | -14 |
| 588 | 17Networks_RH_TempPar_1 | 55 | -2 | -11 |
| 589 | 17Networks_RH_TempPar_2 | 49 | -17 | -10 |
| 590 | 17Networks_RH_TempPar_3 | 49 | -25 | -5 |
| 591 | 17Networks_RH_TempPar_4 | 60 | -14 | -4 |
| 592 | 17Networks_RH_TempPar_5 | 50 | -35 | 3 |
| 593 | 17Networks_RH_TempPar_6 | 61 | -25 | 2 |
| 594 | 17Networks_RH_TempPar_7 | 60 | -45 | 6 |
| 595 | 17Networks_RH_TempPar_8 | 56 | -54 | 10 |
| 596 | 17Networks_RH_TempPar_9 | 51 | -41 | 12 |
| 597 | 17Networks_RH_TempPar_10 | 57 | -47 | 13 |
| 598 | 17Networks_RH_TempPar_11 | 64 | -35 | 11 |
| 599 | 17Networks_RH_TempPar_12 | 62 | -40 | 20 |
| 600 | 17Networks_RH_TempPar_13 | 54 | -44 | 23 |

1. Wagner HH, Dray S, O'Hara RB. Generating Spatially Constrained Null Models for Irregularly Spaced Data Using Moran Spectral Randomization Methods. *Methods in Ecology and Evolution* (2015) 6(10):1169-78. doi: 10.1111/2041-210x.12407.

2. Vos de Wael R, Benkarim O, Paquola C, Lariviere S, Royer J, Tavakol S, et al. Brainspace: A Toolbox for the Analysis of Macroscale Gradients in Neuroimaging and Connectomics Datasets. *Commun Biol* (2020) 3(1):103. Epub 20200305. doi: 10.1038/s42003-020-0794-7.

3. Whitaker KJ, Vértes PE, Romero-Garcia R, Váša F, Moutoussis M, Prabhu G, et al. Adolescence Is Associated with Genomically Patterned Consolidation of the Hubs of the Human Brain Connectome. *Proc Natl Acad Sci U S A* (2016) 113(32):9105-10. Epub 20160725. doi: 10.1073/pnas.1601745113.
